# SET Domain Containing 2 Deficiency in Myelodysplastic Syndrome

**DOI:** 10.3389/fgene.2020.00794

**Published:** 2020-08-06

**Authors:** Jiaming Li, Zhenping Peng, Fangxiu Luo, Yu Chen

**Affiliations:** ^1^Department of Hematology, Ruijin Hospital, Shanghai Jiao Tong University School of Medicine, Shanghai, China; ^2^Department of Clinical Laboratory, Ruijin Hospital, Shanghai Jiao Tong University School of Medicine, Shanghai, China; ^3^Department of Pathology, Ruijin Hospital, Shanghai Jiao Tong University School of Medicine, Shanghai, China

**Keywords:** gene mutation, myelodysplastic syndrome, progression-free survival, SET domain containing 2, *DVL3*

## Abstract

Recent studies have shown that myelodysplastic syndrome’s (MDS) progression to acute myeloid leukemia (AML) is associated with gene mutations. SET domain containing 2 (*SETD2*) variants were reported as a risk factor of poor prognosis in patients with AML. However, little is known about the potential contribution of the *SETD2* gene in MDS. In this study, we investigated the roles of *SETD2* gene mutations/variants on clinical features and prognosis in patients with MDS. A 43-gene panel was used for next-generation sequencing in 203 patients with primary MDS, and then the effects of *SETD2* mutation on Wnt/β-catenin signaling was investigated during the different stages of MDS. At a median follow up of 33 months, 65 (32.0%) deaths and 94 (46.3%) leukemic transformations were recorded. The most frequent mutations/variants included *TET2*, *DNMT3A*, and *ASXL1* mutations/variants. 37 patients had *SETD2* gene mutations/variants, and these patients exhibited a significantly increased frequency of *TP53* mutations. Multivariate survival analyses indicated that *SETD2* mutations/variants were closely associated with overall survival (OS), and they were identified as risk factors for progression-free survival (PFS), especially with low expression of *SETD2* gene. Further, we found that *SETD2* loss could promote MDS progression via upregulation *DVL3* mRNA level in BM cells and it could also cause genomic instability. Secondary mutations, such as *TP53* and *FLT3* mutations, were acquired at the time of progression to AML. In conclusion, we showed that *SETD2* deficiency was associated with poor outcomes in patients with MDS. Moreover, *SETD2* deficiency may upregulate *DVL3* expression and modulate genomic stability that caused AML transformation.

## Introduction

Myelodysplastic syndrome (MDS) is a clonal myeloproliferative disorder of hematopoietic stem cells (HSCs) and can evolve into aggressive forms of acute myeloid leukemia (AML) (Greenberg et al., 2017). Transformation to AML often involves genetic mutations that can be consistently recognized in MDS. Up to 80% of patients with MDS have one or more gene mutations, and as the number of oncogenic mutations increases, overall survival (OS) and progression-free survival (PFS) progressively worsen (Kim et al., 2017; Tefferi et al., 2017). Some of these molecular markers can be used to predict clinical outcomes in patients with MDS (Visconte et al., 2019; Hospital and Vey, 2020). Epigenetic modifications, particularly aberrant methylation of cancer-related genes such as Tet methylcytosine dioxygenase 2 (*TET2*) and DNA methyltransferase 3A (*DNMT3A*), are common abnormalities in MDS (Hosono, 2019). The role of epigenetic deregulation has been well-documented and has led to the successful development of epigenetic therapies. Recently, several studies have demonstrated that the methylation of *TET2*, *DNMT3A*, and *DNMT3B* is related to mutations in SET domain containing 2 (*SETD2*), which can drive tumorigenesis by coordinated disruption of the epigenome and transcriptome (Imielinski et al., 2012; Baubec et al., 2015; Tiedemann et al., 2016; Tlemsani et al., 2016).

The tumor suppressor gene *SETD2* is a histone methyltransferase that functions to trimethylated lysine 36 in histone H3. As a transcriptional regulator, *SETD2* has been shown to participate in diverse biological processes including alternative splicing, transcriptional elongation, DNA repair, and embryonic differentiation (Venkatesh et al., 2012; Li et al., 2013; Neri et al., 2017). *SETD2* mutations are often present and predict poor survival in several types of leukemia as well as various solid tumors (Kandoth et al., 2013; Mar et al., 2014; Zhu et al., 2014; González-Rodríguez et al., 2020). A recent study confirms that loss-of-function *SETD2* mutations facilitate the initiation of leukemia and impair DNA damage recognition, leading to resistance to therapy (Sheng et al., 2019). Another study demonstrate that *SETD2* is required for the self-renewal of HSCs and that *SETD2*-deficient HSCs contribute to the development of MDS (Zhang et al., 2018). Nevertheless, the roles of *SETD2* in MDS remain largely unknown. In this study, we investigated the effects of *SETD2* gene mutations/variants on clinical features and prognosis in patients with MDS, which provided insights into the roles of *SETD2* in MDS.

## Patients and Methods

### Patients

All study participants had been diagnosed with MDS according to World Health Organization criteria (Arber et al., 2016). Patients with MDS identified at Ruijin Hospital North, Shanghai Jiao Tong University from May 2015 to December 2019. This report included follow-up data through March 1, 2020, with a median follow-up period of 33 months (range: 3–60 months). OS and PFS were evaluated as disease outcomes, and events were defined as any AML transformation or death. The OS time was calculated from the time of diagnosis to the time of death or to the last follow-up. PFS was defined as the period beginning when the patient was diagnosed with MDS until the time of AML transformation progression, relapse, or death. Informed consent was obtained from all patients, and the study protocol was approved by the Ethic Committees of Ruijin Hospital North, Shanghai Jiao Tong University School of Medicine.

### Sample Collection and DNA Extraction

Bone marrow (BM) samples were harvested from all patients and patient-matched germline reference samples such as oral mucosal cells, hair with hair follicles, or peripheral blood lymphocytes (PBMC) were also harvested. BM mononuclear cells were obtained by centrifugation on a Ficoll-Hypaque at a density gradient of 1500 × g for 25 min, and then washed three times in phosphate-buered saline (PBS). Next, 1 mL of TRIzol reagent (Invitrogen, Carlsbad, CA, United States) was added, and samples were stored at −20°C. Normal DNA was obtained from normal tissues or blood samples. Blood DNA was extracted by Qiagen blood extraction kit (Qiagen, Hilden, Germany), and tissue DNA was extracted using FastPure FFPE DNA Isolation Kit (Vazyme, Nanjing, China) following the manufacturer’s protocol. DNA Sanger sequencing from patient-matched tissues and PBMC was applied to determine the presence of germline mutations. DNA quality was assessed by agarose gel electrophoresis and NanoDrop 2000 spectrophotometer (Thermo Fisher Scientific, Wilmington, DE, United States).

### Targeted Next-Generation Sequencing

Targeted Sequencing was performed with the illumina Hiseq Xten platform at the sequencing laboratory of Tissuebank Precision Medical Co, Ltd. (Shanghai, China). A total of 10 ng DNA per sample was amplified by PCR, and then the library was captured by using xGen^®^ Lockdown^®^ probes and xGen Hybridization and Wash Kit; Illumina Hiseq sequencer carried out pair end sequencing with a depth of 200X. 43 pathogenic genes (*ASXL1*, *BCOR*, *BCORL1*, *BRAF*, *CALR*, *CBL*, *CDKN2A*, *CEBPA*, *CREBBP*, *CSF3R*, *CUX1*, *DNMT3A*, *ETV6*, *EZH2*, *FLT3*, *GATA1*, *GATA2*, *GNAS*, *IDH1*, *IDH2*, *IKZF1*, *JAK2*, *JAK3*, *KIT*, *KRAS*, *MPL*, *NF1*, *NPM1*, *NRAS*, *PHF6*, *PIGA*, *PTEN*, *PTPN11*, *RUNX1*, *SETBP1*, *SF3B1*, *SRSF2*, *STAG2*, *TET2*, *TP53*, *U2AF1*, *WT1*, *ZRSR2*) were screened in all patients, including the entire coding regions and exon-intron boundaries. This multi-gene panel was expected to cover 100% of the targeted area. DNA was sheared into short genetic fragments (150∼200 bp) using the Covaris LE220, which included purified and captured gene fragments. Adaptor-ligated amplicons were prepared using the Illumina Paired-End Sample Preparation kit. Illumina multi-PE-adaptors were bound to terminal genes and target enrichment was performed by probe capture, amplicons were purified using VAHTS DNA Clean Beads and captured on the Illumina Hiseq Xten instrument.

### Mutation Analysis

Both VarScan and GATK software were adopted for data analysis, including quality assessment, reading comparison, variant identification, variant annotation, visualization, and prioritization. Variant Call Format (VCF) files were annotated with ANNOVAR software, and variants were prioritized using their minor allele frequency of the variant (MAF < 0.01), zygosity, function, location within the gene, and pathogenicity according to ClinVar. MAF was evaluated by data from 1,000 Genomes Project, the Exome Sequencing Project, and the Exome Aggregation Consortium Database. The nature of novel gene mutations was established based on the American College of Medical Genetics and Genomics (ACMG) guideline.

The conservation and deleteriousness of the variants were investigated using ANNOVAR which interrogated the following tools: SIFT, PolyPhen 2 HVAR, Polyphen2 HDIV, MutationTaster, MutationAssessor, Likelihood ratio test (LRT), FATHMM, MetaSVM, MetaLR, GERP++, PhyloP, VEST3, DANN, CADD, PROVEAN, fathmm-MKL, Integrated_fitCons, SiPhy_29way, and PhastCons. Non-synonymous germline mutations with a frequency > 1% or synonymous gene mutations were filtered out. On basis of a combination of these tools (two of the 19 tools predicting damaging effects) to evaluate potential pathogenic gene mutations/variants, we searched the published literature for selected gene mutation/variant studies to further assess their potential pathogenicity. Variants that meet these criteria and do not exist in the control group were considered destructive. Briefly, altered DNA sequences were deemed as mutations/variants if they were associated with a hematologic malignancy, if they were assessed with potential pathogenicity, or if they were suspected to be related to clinical efficacy and safety.

During the analysis, we used genome-wide association studies (GWAS) and cancer genome Atlas (TCGA) to discover germline and somatic mutant genes related to MDS. Somatic gene mutations were identified by comparing paired tissue and BM. We utilized variant calls from non-tumor control samples to filter germline gene mutations, and blood samples to track the VAF of gene mutations. If germline gene mutations were recognized in an individual with MDS, sanger sequencing would be used to screen other available family members to find the identified gene mutations.

### Western Blotting

Total cellular protein was extracted with RIPA lysis buffer (Beyotime; cat. no. P0013B). Protein concentrations were determined using BCA assays. Next, 30 μg protein lysate was separated by sodium dodecyl sulfate/polyacrylamide gel electrophoresis and transferred electrophoretically to polyvinylidene fluoride membranes (Millipore, Bedford, MA, United States). Membranes were immunoblotted with primary antibodies and then horseradish peroxidase-conjugated secondary antibodies in PBS containing 0.5% Tween-20 and 5% bovine serum albumin. The following antibodies were used in this study: anti-SETD2 (Santa Cruz Biotechnology, Santa Cruz, CA, United States; cat. no. sc-99451) and anti-β-catenin (Abways; cat. no. CY3523). Western blot signals were obtained by detecting chemiluminescence on a Typhoon FLA 9500 (GE Healthcare, WI, United States). Image J was used to analyze the signal intensities. Each blot shown in the figures was representative of at least three experiments.

### Immunofluorescence Analysis

Immunofluorescence analysis was performed using standard procedures. Briefly, cells seeded in 24-well plates were fixed with 4% paraformaldehyde and then permeabilized with 1% Triton. Cells were then incubated overnight at 4°C with anti-SETD2 antibodies (Sigma Aldrich, St. Louis, MO, United States; cat. no. HPA042451) or anti-β-catenin antibodies (Abways; cat. no. CY3523) and then detected the next day with AlexaFluor 647 goat anti-mouse IgG antibodies or AlexaFluor 488 alpaca anti-rabbit IgG antibodies. 4′, 6-Diamidino-2-phenylindole was used to stain the nuclei. Immunofluorescence images were observed on a fluorescence microscope (Leica; cat. no. DMI4000B).

### RNA Extraction and Reverse Transcription Quantitative Real-Time Polymerase Chain Reaction (RT-qPCR)

Total RNA was extracted using RNAiso Plus reagent (Takara, Shiga, Japan), and 1.5 μg total RNA from cultured cells was reverse transcribed using a PrimeScriptP RT Reagent Kit (Takara) according to the manufacturer’s instructions. RT-qPCR was performed using a 7500 Fast Real-Time PCR System (Applied Biosystems, Foster City, CA, United States). The amplified transcript level of each specific gene was normalized to that of actin.

### Statistical Analysis

This was a retrospective study, and descriptive statistics were collected at initial diagnosis. Comparison of age and blast cells was analyzed with Student’s *t*-test. Hemoglobin difference was analyzed with Mann-Whitney test. The result of *SETD2* mRNA expression was analyzed by Student’s t test and a chi-squared test after testing for normality with the Kolmogorov-Smirnov test. Categorical variables were compared using Fisher’s exact test or chi-squared test as appropriate. Patient groups with nominal variables were compared by chi-squared test. Survival analysis was considered from the date of diagnosis to date of death or last contact. Survival curves were prepared by the Kaplan-Meier method and compared by the log-rank test. Cox proportional hazards regression model and multivariate cox proportional hazards models were used to calculate hazard ratios (HRs) with 95% confidence intervals (CIs) of association pertaining to the relationship between risk factors and survival. Statistical analyses were conducted with SPSS software, version 21.0. Statistical significance was determined by log-rank test, chi-squared test, or Fisher’s exact test. For all statistical tests, a *P*-value of less than 0.05 was considered significant.

## Results

### Patient Cohort: Clinical Characteristics

In total, 203 patients with primary MDS, including 137 men and 66 women, were enrolled in this study. The median age at diagnosis was 60 years (range: 30–80 years). The IPSS-R risk distribution was 15.8% very high, 26.1% high, 36.5% intermediate, and 21.7% low. The median bone marrow blasts and hemoglobin were 7% (range: 0.5–19.0%) and 65 g/L (range: 36–109 g/L). OS and PFS were evaluated as disease outcomes, and events were defined as any AML transformation or death. All survival end points were censored at the date of last follow-up when progression or death was not observed. During follow-up, 65 (32.0%) deaths and 94 (46.3%) leukemic transformations were recorded. Patients received treatment with hypomethylating agents (*n* = 182), induction chemotherapy (*n* = 87), allogeneic stem cell transplantation (*n* = 14), and lenalidomide/thalidomide/danazol (*n* = 10).

### Gene Mutations in MDS

At least one mutation/variant was detected in 166 (81.8%) patients; 36.5% harbored three or more mutations/variants. The most frequentmutations/variants included *TET2* (26.1%), *DNMT3A* (19.2%), *ASXL1* (18.2%), *SETD2* (18.2%), SRSF2 (14.8%), *TP53* (13.3%), *SF3B1* (10.8%), *U2AF1* (14.3%). The common gene mutations/variants weredetected in *RUNX1* (5.4%), *IDH2* (4.4%), *SETBP1* (3.4%), *JAK2* (4.9%), *CBL* (3.9%), *CEBPA* (3.0%), *ETV6* (2.5%), *IDH1* (1.5%) and *CSF3R* (1.0%). *SETD2* mutations/variants werefound in 37 patients (18.2%), including eight single nucleotidevariants: p.(M761I), *n* = 1; p.(E639K), *n* = 2; p.(P193L), *n* = 2; p.(M1080I), *n* = 7; p.(N1155K), *n* = 15; p.(P1962L), *n* = 28; p.(L2486R), *n* = 1; p.(E1142G), *n* = 1; and two frameshift mutations [p.(T2388fs), *n* = 1; p.(F1116fs), *n* = 1]. *SETD2* p.(P1962L) (13.8%), p.(N1155K) (7.4%) and p.(M1080I) (3.4%) were more common in patients with MDS. We evaluated the relationships between *SETD2* and other gene mutations/variants, and found that 37 patients with *SETD2* alterations had at least one other alterations. Notably, they showed significantly more frequent *TP53* gene mutations compared with patients with wild-type *SETD2* (37.8% vs. 7.8%, respectively; *P* < 0.001). Moreover, *SETD2* mutations/variants were associated with higher BM blast content (10% vs. 6%, respectively; *P* < 0.001) and death rates (59.5% vs. 25.9%, respectively; *P*< 0.001). The clinical characteristics of patients with *SETD2* mutations/variants were summarized in Table 1.

**TABLE 1:** Characteristics of patients according to SETD2 mutation status.

Characteristics	*SETD2* mutations/variants *n* = 37	*SETD2* wide type *n* = 166	*P*-value
Age in years, median (range)	62 (33–80)	60 (30–79)	0.177
Hemoglobin, g/L, median (range)	65 (36–99)	67 (36–109)	0.293
BM blast %, median (range)	10 (1–19)	6 (0.5–19)	<0.001
IPSS-R, n (%)			0.173
Very high	4 (10.8%)	28 (16.9%)	
High	12 (32.4%)	41 (24.7%)	
Intermediate	17 (46.0%)	57 (34.3%)	
Low	4 (10.8%)	40 (24.1%)	
*TP53* Mutation, n (%)	14 (37.8%)	13 (7.8%)	<0.001
*ASXL1* Mutation, n (%)	6 (16.2%)	29 (17.5%)	0.855
Death	22 (59.5%)	43 (25.9%)	<0.001
AML transformation	21 (56.8%)	73 (44.0%)	0.159
OS, months, median (range)	16 (2–60)	22 (1–68)	0.186
PFS, months, median (range)	11 (1–53)	15 (1–64)	0.077

### *SETD2* Mutations/Variants Predicted Poor Prognosis in MDS

*ASXL1* mutations/variants were of no significance to inferior OS (Table 2); *TP53* mutations/variants were related to inferior OS both on univariate analyses [hazard ratio (HR) = 3.5, 95% confidence interval (CI): 1.9–6.2, *P* < 0.0001] and multivariate analyses (HR = 2.7, 95% CI: 1.4–5.0, *P* = 0.003); *SETD2* mutations/variants were also identified as risk factors for inferior OS by both univariate analysis (HR = 2.7, 95% CI: 1.6–4.4, *P* = 0.0002) and multivariable analysis (HR = 2.0, 95% CI: 1.2–3.5, *P* = 0.01). The addition of age risk stratification to the multivariate model did not affect the significance of *SETD2* and *TP53* for inferior OS.

**TABLE 2:** Univariate and multivariate analyses of Overall and Progression-free survival in 203 patients with MDS.

Mutations	Univariate *P-*value; HR (95%CI)	Multivariate *P*-value; HR (95%CI)	Multivariate age adjusted *P*-value; HR (95%CI)
**Overall survival**			
*SETD2*	0.0002; 2.7 (1.6–4.4)	0.01; 2.0 (1.2–3.5)	0.03; 1.9 (1.1–3.3)
*TP53*	*P* < 0.0001; 3.5 (1.9–6.2)	0.003; 2.7 (1.4–5.0)	0.02; 2.2 (1.1–4.2)
*ASXL1*	0.9; 1.0 (0.5–1.8)	0.8; 1.1 (0.6–2.1)	0.9; 1.0 (0.6–2.0)

**Progression-free survival**			

*SETD2*	*0.05*; 1.6 (1.0–2.6)	0.4; 1.2 (0.7–2.1)	0.2; 1.4 (0.8–2.3)
*TP53*	*P* < 0.0001; 2.9 (1.8–4.8)	0.0003; 2.7 (1.6–4.6)	0.02; 1.9 (1.1–3.2)
*ASXL1*	0.6; 0.9 (0.5–1.5)	0.9; 1.0 (0.6–1.6)	0.9; 1.0 (0.6–1.7)

**FIGURE 1:**
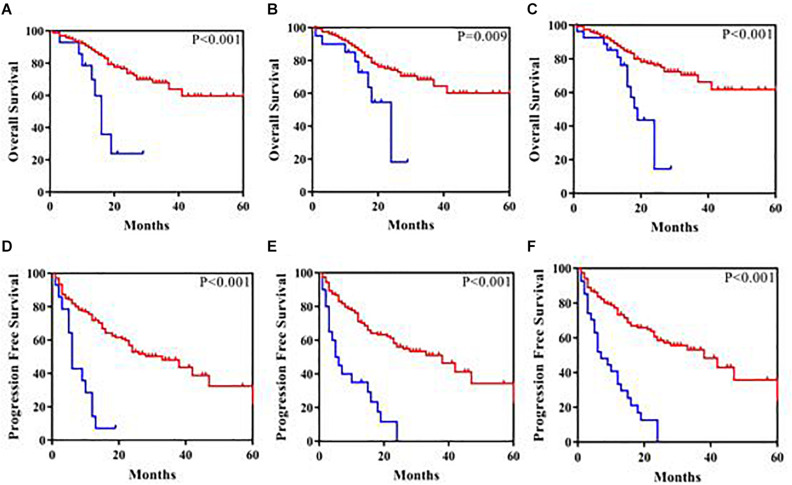
Kaplan-Meier analysis of overall survival and progression-free survival in 180 patients. **(A)** Overall survival according to *SETD2* mutations/variants present (the blue color) compared with *SETD2* mutations/variants absent (the red color). **(B)** Overall survival according to *TP53* mutations/variants present (the blue color) compared with *TP53* mutations/variants absent (the red color). **(C)** Overall survival according to *SETD2* or *TP53* mutations/variants present (the blue color) compared with *SETD2* or *TP53* mutations/variants absent (the red color). **(D)** Progression-free survival according to *SETD2* mutations/variants present (the blue color) compared with *SETD2* mutations/variants absent (the red color). **(E)** Progression-free survival according to *TP53* mutations/variants present (the blue color) compared with *TP53* mutations/variants absent (the red color). **(F)** Progression-free survival according to *SETD2* or *TP53* mutations/variants present (the blue color) compared with *SETD2* or *TP53* mutations/variants absent (the red color).

*ASXL1* mutations/variants were of no significance to inferior PFS (Table 2); *TP53* mutations/variants were related to inferior PFS both on univariate analyses (HR = 2.9, 95% CI: 1.8–4.8, *P* < 0.0001) and multivariate analyses (HR = 2.7, 95% CI: 1.6–4.6, *P* = 0.0003), and the addition of IPSS-R risk stratification to the multivariate model did not affect the significance of *TP53* for inferior PFS. However, *SETD2* mutations/variants were of only borderline significance on univariate analysis (*P* = 0.05). We investigated the conservation and deleteriousness of mutations/variants by using the soft tools (Supplementary Table 1) and searching the published study (Wang et al., 2019), We found *SETD2* p.(P1962L) and p.(N1155K) were not considered as damaging. The variant allele frequency (VAF) of *SETD2* were tracked before and after treatment. Following the decitabine administration, it was shown that the VAF of *SETD2* p.(P1962L) (Sample 55, 21% vs. 38%; Sample 46, 100% vs. 39%; Sample 69, 53% vs. 39%; Sample 37, 60% vs. 45%) and p.(N1155K) (Sample 42, 18% vs. 0%; Sample 44, 100% vs. 0%; Sample 84, 51% vs. 32%; Sample 46, 100% vs. 48%) experienced a marked change, which suggested the likely association with therapy outcome in MDS. Therefore, patients with mutations/variants were divided into two groups [Group A, *n* = 23, only with *SETD2* p.(N1155K) or p.(P1962L); Group B, *n* = 14, the remaining mutations/variants]. The differences were statistically significant between the two groups on PFS (Supplementary Table 2). For Group B, univariate analysis of PFS identified *SETD2* mutations/variants as a significant risk factor (HR = 4.3, 95% CI: 2.3–7.9, *P* <0.0001), and this factor retained significance during multivariate analysis (HR = 3.0, 95% CI: 1.5–5.8, *P* = 0.001). The addition of IPSS-R to the multivariate model did not affect the impact of *SETD2* on PFS (Table 3). In order to better understand the risk-specific prognostic value, we performed additional analyses by grouping *SETD2* or *TP53* together as adverse mutations/variants for PFS. Kaplan-Meier analysis for PFS identified *SETD2* or *TP53* as significant risk factors (*P* < 0.001, Figure 1). However, for Group A, *SETD2* variants were not related to inferior PFS (Supplementary Table 3). The most recent study demonstrated that low expression of *SETD2* promoted the transformation of MDS into AML (Chen et al., 2020), which was consistent with our outcomes of RT-PCR. The pretreatment baseline expression of *SETD2* mRNA (Group A, *n* = 16; Group B, *n* = 14) was lower in the two groups than those in *SETD2* mutations/variants absent controls (Group C, *n* = 20), and there was evident difference between the two groups (Group A vs. Group B, *P* < 0.001) (Supplementary Table 4). In all, *SETD2* mutations/variants were considered as significant risk factor of poor outcomes in MDS patients, especially with low expression of *SETD2* gene.

**TABLE 3:** Univariate and multivariate analyses of Overall and Progression-free survival in 180 patients with MDS.

Mutations	Univariate P value; HR (95%CI)	Multivariate *P*-value; HR (95%CI)	Multivariate age adjusted *P*-value; HR (95%CI)
**Overall survival**			
*SETD2*	0.0005; 3.9 (1.8-8.5)	0.01; 2.9 (1.2–7.0)	0.03; 2.7 (1.1–6.4)
*TP53*	0.009; 2.7 (1.3-5.6)	0.1; 1.9 (0.8–4.5)	0.2; 1.7 (0.7–3.9)
*ASXL1*	0.6; 1.2 (0.6-2.3)	0.5; 1.2 (0.6–2.4)	0.9; 1.1 (0.5–2.1)

**Progression-free survival**			
*SETD2*	*P*< 0.0001; 4.3 (2.3-7.9)	0.001; 3.0 (1.5–5.8)	*P* < 0.0001; 4.3 (2.2–8.5)
*TP53*	*P* < 0.0001; 4.0 (2.4-6.8)	*P* < 0.0001; 3.2 (1.8–5.7)	0.01; 2.1 (1.2–3.6)
*ASXL1*	0.9; 1.0 (0.6-1.6)	1.0; 1.0 (0.6–1.7)	0.9; 0.9 (0.6–1.7)

### *SETD2* May Modulate Wnt Signaling by Regulating *DVL3* Expression

**FIGURE 2:**
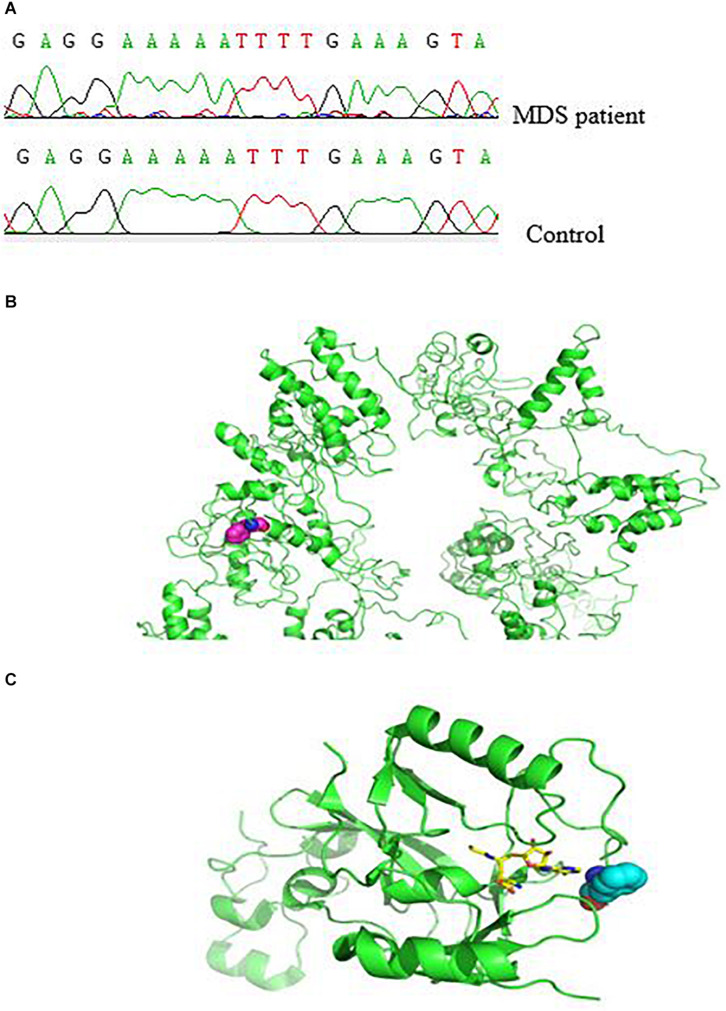
Effect of *SETD2* p. F1116fs in a patient with MDS. **(A)** Part of the *SETD2* gene sequence containing the 3350–3351insT mutation. **(B)** Modeling of the structure of SETD2 protein and location of amino acid mutations. The green color indicates the structure of SETD2 protein, and the red line shows the amino acids changed to terminating codons. **(C)** Locations of amino acid mutations in *SETD2* and functional binding regions. The green color indicates SETD2 protein, the red line shows the amino acids changed to terminating codons, blue lines show functional binding sites, and colored lines show the small molecules. The protein was modeled using I-TASSER and Pymol software.

Next, we characterized the molecular and genetic abnormalities of a novel variant form in a patient with MDS. DNA sequencing analysis showed a homozygous single-base insertion between nucleotides 3350 and 3351 in the *SETD2* coding sequence (Figure 2A). In order to predict and to better understand the functions of the mutated SETD2 protein, the ITASSER server was used to construct the three-dimensional (3-D) structural of this protein (Shanghai Jiao Tong University School of Medicine, Shanghai, China). Protein structural and functional prediction analysis showed this mutation resulted in the deletion of amino acids after amino acid 1116 in SETD2 and the formation of a new truncated SETD2 molecule (Figure 2B); however, the truncated SETD2 lacked functional binding sites, which could not work properly (Figure 2C).

**FIGURE 3:**
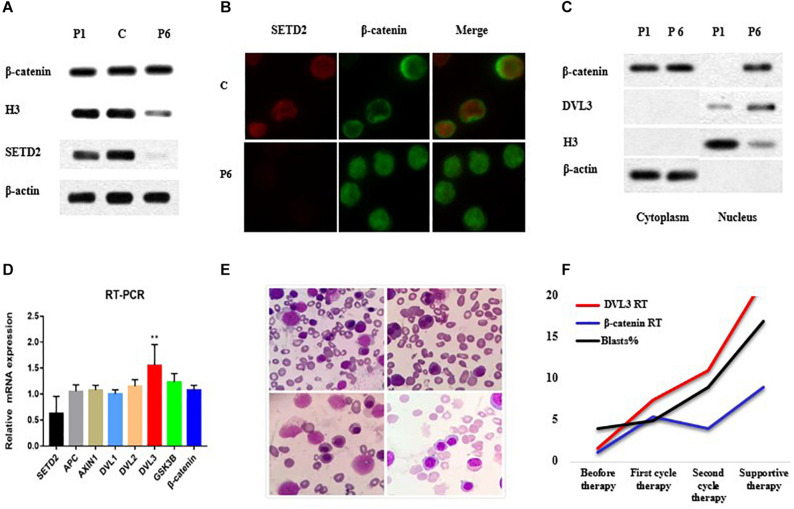
*SETD2* modulated *DVL3* expression to regulate Wnt signaling. **(A–C)** Effects of *SETD2* mutation on the nuclear localization of β-catenin in patients with MDS. **(D)** RT-PCR analysis of Wnt target gene expression in patients with MDS. **(E)** The definitive erythroid development in BM smears. **(F)** The level of gene expression and blasts numbers before and after treatment. C, control; P1, MDS case without *SETD2* mutation; P6, MDS case with *SETD2* p. F1116fs mutation. ^∗∗^*P* < 0.05, the level of *DVL3* mRNA expression was higher than that of control.

Accordingly, we then performed western blotting using BM cells from the patient and showed that SETD2 was almost undetectable when the *SETD2* mutation was identified at initial diagnosis of MDS (Figure 3A). Notably, the distribution of β-catenin in BM cell nuclei was significantly increased, as demonstrated by fluorescence microscopy (Figure 3B). Similarly, subcellular fractionation and western blot analyses of BM cells from case 6 confirmed that *SETD2* expression was deficient in the nucleus, whereas the level of β-catenin in the nucleus was enhanced compared with that in the cytoplasmic control (Figure 3C). RT-qPCR analyses indicated that these BM cells with *SETD2* mutation produced normal levels of *GSK3B*, *APCS*, *DVL1*, and *DVL2* mRNAs, whereas *DVL3* mRNA was upregulated in the absence of *SETD2* (Figure 3D). The patient received decitabine combined with AA regimen (aclarubicin, Ara-C); however, it had no response and transformed to AML after two cycles of therapy. He developed severe anemia, and pathological hematopoiesis was found everywhere in bone marrow smears, suggesting the deficient erythroblast differentiation (Figure 3E). With the increased numbers of blast cells in BM, the level of *DVL3* and β-catenin mRNA expression improved synergistically (Figure 3F), while the protein level of *DVL3* was not increased, suggesting that β-catenin might be indirectly regulated at protein level.

**FIGURE 4:**
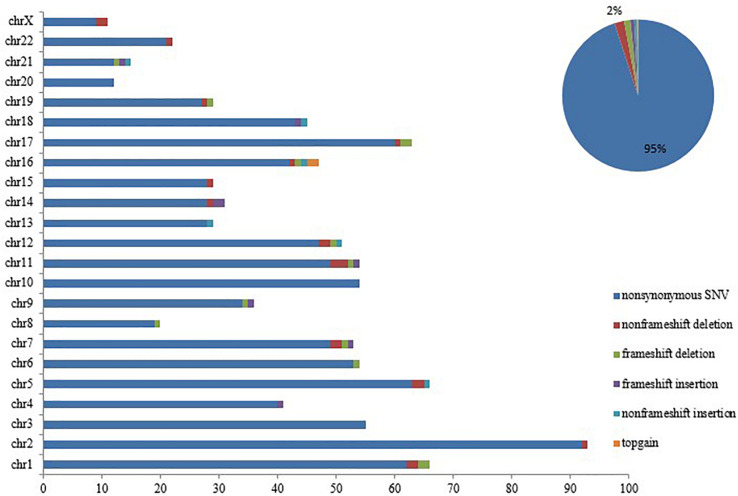
*SETD2* mutation caused genomic instability. Number and type of somatic mutations newly identified in each chromosome when the patient progressed to AML, showing a majority of nonsynonymous variants.

Notably, loss of *SETD2* could cause DNA replication defects and genomic instability. Somatic mutations were successively acquired at the time of progression to AML. These mutations in each chromosome were listed in Figure 4. Number and type of newly identified mutations showed a majority of nonsynonymous variants. It should be noted that the new mutations in genes involved in signaling pathway (*FLT3, TP53, TET2, ASXL1*) were identified when the patient with MDS transformed to AML (Table 4). Additionally, these additional mutations were accompanied by expansion of existing mutations (*TGFβ* p.(P10L), *IL3* p.(P27S), *IL10* p.(R351G). Finally, the patient died of cerebral hemorrhage.

**TABLE 4:** Newly somatic variants identified by whole genome sequencing when MDS progression to AML.

Chromo some	Mutation type	Mutation location	AA change	SIFT	Polyphen2 -HDIV	Polyphen2 -HVAR	Frequency
Chr4	Nonsynonymous SNV	*TET2*:NM_001127208:exon3:c.C86G	p.(P29R)	D	D	P	40%
Chr13	Nonsynonymous SNV	*FLT3*:NM_004119:exon9:c.A1073T	p.(D358V)	T	B	B	63%
Chr13	Nonsynonymous SNV	*FLT3*:NM_004119::exon6:c.C680T	p.(T227M)	T	D	P	59%
Chr17	Nonsynonymous SNV	*TP53*:NM_001126115:exon3:c.C326T	p.(S109F)	D	D	D	100%
Chr20	Frameshift deletion	*ASXL1*:NM_015338:exon12:c.2128delG	p.(G710fs)	D	D	D	50%

## Discussion

In this study, we found that *TET2*, *DNMT3A*, *ASXL1*, and *TP53* were commonly mutated in 203 patients with MDS, consistent with a previous study (Yu et al., 2020). *SETD2* mutations have been detected in a subset leukemia. For example, Non-*MLL* rearranged AML and chronic lymphocytic leukemia exhibit similar incidence rates of *SETD2* mutations (6 and 7%, respectively), and a lower incidence (3%) has been reported in chronic lymphocytic leukemia (Masetti et al., 2016; Parker et al., 2016). *SETD2* mutations/variants were detected in 37 of 203 cases, and new mutations were only found in four cases (2.0%). These studies did identify the rare nature of *SETD2* mutations in leukemia and MDS. *SETD2* mutations/variants often occurred simultaneously with *TP53* mutations in our study. Recently, the *SETD2* gene has been shown to directly regulate the transcription of a subset of genes via cooperation with the transcription factor *TP53* and contribute to further inactivation of *TP53*-mediated checkpoint control (Carvalho et al., 2014; Xie et al., 2008). In addition, *SETD2* mutations have been linked to poor clinical prognosis in various tumors, such as in renal clear cell carcinoma and AML (Wang et al., 2019; González-Rodríguez et al., 2020). Notably, *SETD2* deficiency has been found to impair HSC self-renewal and induce MDS transformation in a conditional *SETD2*-knockout mouse model (Zhang et al., 2018). In our study, we assessed the effects of *SETD2* mutations/variants in patients with MDS, and observed that *SETD2* deficiency was an IPSS-R-independent factor predicting shorter PFS in both univariate and multivariate analyses.

Evidence from human genomes sequencing has linked *SETD2* to MDS, but its causal role has not been reported yet. Previous observation was that *SETD2* gene modulated Wnt signaling by regulating β-catenin (Yuan et al., 2017), and S*ETD2* could enhance susceptibility to tumorigenesis in the context of dysregulated Wnt signaling through epigenetic regulation of RNA processing, including *DVLs* (Barry et al., 2013; Wang et al., 2015). Interestingly, our results indicated that *SETD2* loss could promote MDS progression via upregulation of *DVL3* in a patient harboring *SETD2* p.(F1116fs) mutation. This finding seemed to be different from previous research. Sun group reported that *SETD2* loss did not affect Wnt/β-catenin signaling in pancreatic ductal adenocarcinoma cells in the context of Kras^G12D9^ (Niu et al., 2020). Yuan group demonstrated that *SETD2* regulated the Wnt pathway indirectly by altering splicing of *DVL2* (Yuan et al., 2017). Given the above research results, we hypothesized that *SETD2* loss can cooperate with other driver mutations to regulate Wnt/β-catenin signaling in the development of MDS. In order to find some clues about the secondary mutations that cause AML transformation, we performed a whole genome sequence of BM cells from the patient at different stages of disease. *ASXL1* and *TET2* mutations were newly detected; non-histone targets of *STED2* also have been found, such as *TP53* and *FLT3* mutations, which implicated a vital role in cell cycle signaling. Despite many additional complicated factors, including the patient receiving decitabine treatment and the discovery of new genetic mutations, it was indicated that *DVL3* was the major isoform among *DVLs* in MDS.

Was there any other mechanism involved to mediate the function of *SETD2* in the transformation from MDS to AML? It was unclear. First, the incidence of *SETD2* gene mutations was low, and we couldn’t observe the up-regulation of *DVL3* gene by *SETD2* from other patients. Secondly, due to the scarcity of primary tumor cells, we were not able to further study the *SETD2* gene on epigenetic regulation of RNA processing. Finally, given the evolution of cloned genes, the role of synergistic genes in regulating Wnt signaling pathways couldn’t be clearly defined. We only described this phenomenon that *SETD2* modulated Wnt signaling by regulating *DVL3* expression in a patient with MDS. Next, it would be necessary to further verify the regulation mechanism of *SETD2* on Wnt signaling pathway in *SETD2* gene knockout mouse model.

In summary, we demonstrated that *SETD2* alterations were associated with worse PFS in Chinese patients with MDS, in addition, *SETD2* loss may modulate genomic stability and upregulate *DVL3* expression through Wnt/β-catenin signaling. Although additional studies are needed to elucidate the biological importance of *SETD2* mutations in MDS, our data provided insights into the role of *SETD2* in MDS and suggested that this gene may be a novel therapeutic target in MDS, as well as other human cancers with *SETD2* deficiency.

## Data Availability Statement

The datasets presented in this study can be found in online repositories. The names of the repository/repositories and accession number(s) can be found in the article/ Supplementary Material.

## Ethics Statement

The studies involving human participants were reviewed and approved by the ethics committee of Ruijin Hospital North, Shanghai Jiao Tong University School of Medicine. The patients/participants provided their written informed consent to participate in this study.

## Author Contributions

FL proposed the concept of the study and was involved in the patient’s clinical characterization. JL and ZP performed the patient’s genetic analysis. YC was involved in all steps of the functional study. JL and FL prepared the manuscript. All authors critically reviewed the manuscript.

## Conflict of Interest

The authors declare that the research was conducted in the absence of any commercial or financial relationships that could be construed as a potential conflict of interest.
