# Supplementary material for: SET Domain Containing 2 Deficiency in Myelodysplastic Syndrome
Source: Front Genet. 2020 Aug 6;11:794. doi: 10.3389/fgene.2020.00794 (PMC7423969; doi:10.3389/fgene.2020.00794)

TABLE1 Gene prediction analysis of 37 patients with *SETD2* mutations/variants

Mutation type	Sample n (%)	Location	AAchange	SIFT	Polyphen2_ HDIV_pred	Polyphen2_H VAR_pred	FATHMM _pred	Somatic varians	Frequency
SNV	7(3.4%)	c.G3240A	p.(M1080I)	D	B	B	Damaging	Yes	45%-100%
SNV	1(0.5%)	c.G2283A	p.(M761I)	T	B	B	Damaging	Yes	63%
SNV	2(1.0%)	c.G1915A	p.(E639K)	D	D	D	Damaging	Yes	70%/90%
SNV	1(0.5%)	c.G3424C	p.(E1142G)	D	B	B	Damaging	Yes	78%
nonframeshift	1(0.5%)	c.3350-3351insT	p.(F1116fs)	-	-	-	n/a	Yes	100%
SNV	1(0.5%)	c.T7457G	p. (L2486R)	D	D	D	Damaging	Yes	54%
SNV	2(1.0%)	c.C578T	p.(P193L)	D	D	D	Damaging	Yes	97%/100%
nonframeshift	1(0.5%)	c.7162delA	p.(T2388fs)	-	-	-	n/a	Yes	57%
SNV	28(13.8%)	c.C5885T	p.(P1962L)	T	B	B	n/a	Yes	23%-100%
SNV	15(7.4%)	c.T3465C	p.(N1155K)	T	B	B	n/a	Yes	37%-100%

TABLE2 Characteristics of 37 patients by *SETD2* vriants status

Characteristics	Group A n=23	Group B n=14	P Value
Age in years, median (range)	60(33-80)	63(55-71)	0.179
Hemoglobin, g/L, median (range)	69(50-109)	60(43-99)	0.398
BM blast %, median (range)	7(0.5-17)	10(3-19)	0.699
IPSS-R, n (%)			0.704
Very high	2(8.7%)	2(14.3%)	
High	8(34.8%)	4(28.6%)	
Intermediate	10(43.5%)	7(50.0%)	
Low	3(13.0%)	1(7.1%)	
Death	14(60.9%)	8(57.1%)	
AML transformation	8(34.8%)	13(92.9%)	0.001
OS, months, median (range)	23(2-60)	13.5(3-29)	0.009
PFS, months, median (range)	15(1-53)	6(1-19)	0.002

TABLE 3 Univariate and multivariable analysis of Overall and Progression-free survival in 189 MDS patients

Overall survival			
Mutations	Univariate<br>P value; HR (95%CI)	Multivariate<br>P value; HR (95%CI)	Multivariate age adjusted<br>P value; HR (95%CI)
SETD2	0.01; 2.2(1.2-4.0)	0.09; 1.7(0.9-3.3)	0.2;1.6(0.8-3.1)
TP53	0.0001; 3.5(1.8-6.7)	0.002; 3.0(1.5-6.0)	0.01;2.4(1.2-5.0)
ASXL1	0.8; 0.9(0.5-1.8)	0.9; 1.1(0.5-2.1)	1.0; 1.0(0.5-2.0)
Progression-free survival			
Mutations	Univariate<br>P value; HR (95%CI)	Multivariate<br>P value; HR (95%CI)	Multivariate IPSS-R adjusted<br>P value; HR (95%CI)
SETD2	0.6;0.8(0.4-1.7)	0.3;0.6(0.3-1.4)	0.3;0.7(0.3-1.4)
TP53	0.006;2.3(1.3-4.2)	0.003;2.6(1.4-4.8)	0.1; 1.6(0.9-3.1)
ASXL1	0.6; 0.9(0.5-1.5)	0.8; 0.9(0.5-1.6)	0.7; 0.9(0.5-1.6)

TABLE 4 The mRNA expression levels of *SETD2*

GroupB		GroupA		GroupC		Controls	
Sample （n=14）	*SETD2* $2^{-\Delta\Delta CT}$	Sample （n=16）	*SETD2* $2^{-\Delta\Delta CT}$	Sample （n=20）	*SETD2* $2^{-\Delta\Delta CT}$	Sample （n=10）	*SETD2* $2^{-\Delta\Delta CT}$
177	0.70	123	1.00	53	0.42	C0	1.00
175	0.24	42	1.38	106	0.54	C1	1.10
176	0.42	59	1.70	60	0.53	C2	1.10
178	0.36	58	1.36	93	2.21	C3	0.94
55	0.73	4-C	1.27	98	2.29	C4	0.90
37	0.34	84	1.32	181	1.14	C5	1.01
194	0.57	138	0.36	154	1.20	C6	1.03
160	0.55	139	1.00	63	0.80	C7	1.15
92	0.84	46	1.29	170	1.67	C8	0.92
10	0.88	174	1.70	131	1.34	C9	1.13
31	0.30	69	1.55	33	0.78		
100	0.89	57	0.69	14	2.57		
52	0.78	60	0.69	132	1.39		
89	0.80	62	0.72	23	1.70		
		105	0.43	201	2.60		
		74	1.40	19	1.30		
				22	0.90		
				65	0.65		
				86	0.77		
				120	0.70		

Group A, n=16, MDS patients only with SETD2 p.(N1155K) or p.(P1962L) variants; *Group B, n=14, MDS patients with the remaining SETD2 mutations/variants)*; *Group C, n=20, MDS patients with SETD2 mutations/variants absent*; *Controls, n=10, normal control.*

MATERIALS 1 Gene mutations/variants in 203 MDS cases

SAMPLE	SETD2 mutations/variants	Gene Mutations/Variants
177	exon3:c.3350-3351insT:p.F1116fsX	TP53:NM_000546:exon7:c.C722T:p.S241F<br>DNMT3A:NM_022552:exon23:c.G2645A:p.R882H<br>SRSF2:NM_003016:exon1:c.C284A:p.P95H<br>SF3B1:NM_012433:exon14:c.G1998T:p.K666N
175	exon3:c.G1915A:p.E639K	TP53:NM_000546:exon4:c.C215G:p.P72R
176	exon3:c.G1915A:p.E639K	TP53:NM_000546:exon8:c.G818T:p.R273L
178	exon3:c.G3424C:p.E1142G	DNMT3A:NM_022552:exon19:c.G2207A:p.R736H<br>SRSF2:NM_003016:exon1:c.C284A:p.P95H<br>SF3B1:NM_012433:exon14:c.G1998T:p.K666N
158		Wild type
115		TET2: NM_001127208:exon6:c.3750_3751insCTT:p.E1250delinsEL<br>SF3B1：NM_012433:exon15:c.A2098G:p.K700E
123	exon12:c.C5885T:p.P1962L	TET2:NM_001127208:exon3:c.C2191T:p.Q731X<br>TP53:NM_000546:exon8:c.G818T:p.R273L<br>SF3B1:NM_012433:exon14:c.G1874T:p.R625L
85-C		ASXL1:NM_015338:exon12:c.1927dupG:p.G642fs<br>PHF6:NM_001015877:exon7:c.T724C:p.C242R<br>U2AF1:NM_006758:exon6:c.A470C:p.Q157P
166		RUNX1:NM_001754:exon6:c.G611A:p.R204Q
188		DNMT3A:NM_022552:exon23:c.G2645A:p.R882H<br>SRSF2:NM_003016:exon1:c.284_307del:p.95_103del<br>SF3B1：NM_012433:exon15:c.A2098G:p.K700E
125		Wild type
107		TET2:NM_001127208:exon11:c.A5284G:p.I1762V<br>TET2:NM_001127208:exon3:c.1867delA:p.K623fs
42	exon12:c.C5885T:p.P1962L<br>exon3:c.T3465C:p.N1155K	TET2:NM_001127208:exon11:c.C4636T:p.Q1546X<br>ASXL1:NM_015338:exon12:c.1927dupG:p.G642fs<br>SRSF2:NM_003016:exon1:c.284_307del:p.95_103del<br>ETV6:NM_001987:exon3:c.G196A:p.V66I
150		JAK2:NM_004972:exon14:c.G1849T:p.V617F<br>PIGA:NM_002641:exon2:c.313delA:p.T105fs
59	exon12:c.C5885T:p.P1962L	CEBPA:NM_004364:exon1:c.97_119del:p.F33fs
58	exon12:c.C5885T:p.P1962L<br>exon3:c.T3465C:	CSF3R:NM_156039:exon14:c.C1853T:p.T618I<br>ASXL1:NM_015338:exon12:c.1971dupG:p.E657fs<br>RUNX1:NM_001754:exon6:c.G611A:p.R204Q

	p.N1155K	
44	exon12:c.C5885T:p.P1962L exon3:c.T3465C:p.N1155K	NPM1:NM_002520:exon11:c.860_861insCTGC:p.L287fs<br>DNMT3A:NM_022552:exon23:c.G2645A:p.R882H<br>FLT3:NM_004119:exon20:c.G2503T:p.D835Y
53		TP53:NM_000546:exon8:c.C817T:p.R273C<br>TP53:NM_000546:exon6:c.A659G:p.Y220C<br>TET2:NM_001127208:exon3:c.2816dupA:p.Q939fs
4-C	exon12:c.C5885T:p.P1962L exon3:c.T3465C:p.N1155K	JAK2:NM_004972:exon14:c.G1849T:p.V617F<br>TET2:NM_001127208:exon11:c.C5681G:p.P1894R<br>SRSF2:NM_003016:exon1:c.C284A:p.P95H
93		RUNX1:NM_001754:exon5:c.494_495insGG:p.G165fs
98		TET2:NM_001127208:exon3:c.G652A:p.V218M<br>RUNX1:NM_001754:exon6:c.548_549insCC:p.P183fs
84	exon12:c.C5885T:p.P1962L exon3:c.T3465C:p.N1155K	KRAS:NM_004985:exon3:c.G179A:p.G60D<br>ASXL1:NM_015338:exon12:c.2128delG:p.G710fs<br>SRSF2:NM_003016:exon1:c.C284T:p.P95L<br>NRAS:NM_002524:exon2:c.G34T:p.G12C<br>CBL:NM_005188:exon9:c.G1259A:p.R420Q<br>SETBP1:NM_015559:exon4:c.G2602A:p.D868N
171		Wild type
181		DNMT3A:NM_022552:exon23:c.G2645A:p.R882H<br>SRSF2:NM_003016:exon1:c.284_307del:p.95_103del
154		Wild type
63		FLT3:NM_004119:exon20:c.G2503T:p.D835Y<br>TET2:NM_001127208:exon11:c.C5482T:p.Q1828X<br>TET2:NM_001127208:exon3:c.1495delC:p.P499fs<br>ASXL1:NM_015338:exon12:c.2761_2762insCCAGAGAACA:p.S921fs<br>JAK2V617F<br>ASXL1:NM_015338:exon12:c.2763_2767del:p.S921fs
55	exon12:c.C5885T:p.P1962L exon3:c.T3465C:p.N1155K exon3:c.G3240A:p.M1080I	RUNX1:NM_001754:exon9:c.1210dupC:p.H404fs<br>ASXL1:NM_015338:exon12:c.1927dupG:p.G642fs<br>SRSF2:NM_003016:exon1:c.C284T:p.P95L<br>CBL:NM_005188:exon8:c.G1223C:p.W408S<br>TET2:NM_001127208:exon3:c.C2113T:p.Q705X
170		Wild type
131		Wild type
157		Wild type

37	exon12:c.C5885T:p.P1962L exon3:c.G3240A:p.M1080I	SETBP1:NM_015559:exon4:c.G2602A:p.D868N NRAS:NM_002524:exon2:c.G34T:p.G12C NRAS:NM_002524:exon2:c.G34A:p.G12S
135	exon12:c.C5885T:p.P1962L	SETD2
27		ASXL1:NM_015338:exon12:c.2683_2684del:p.S895fs. DNMT3A:NM_022552:exon23:c.G2645A:p.R882H IDH2:NM_002168:exon4:c.G419A:p.R140Q RUNX1:NM_001754:exon8:c.C958T:p.R320X. SRSF2:NM_003016:exon1:c.C284A:p.P95H.
180		Wild type
134		Wild type
138	exon12:c.C5885T:p.P1962L exon3:c.T3465C:p.N1155K	TP53:NM_001126116:exon2:c.A263G:p.Y88C,TP53:NM_001126117:exon2:c.A263G:p.Y88C,TP53:NM_001276697:exon2:c.A182G:p.Y61C,TP53:NM_001276698:exon2:c.A182G:p.Y61C,TP53:NM_001276699:exon2:c.A182G:p.Y61C,TP53:NM_001126118:exon5:c.A542G:p.Y181C,TP53:NM_000546:exon6:c.A659G:p.Y220C,TP53:NM_001126112:exon6:c.A659G:p.Y220C,TP53:NM_001126113:exon6:c.A659G:p.Y220C,TP53:NM_001126114:exon6:c.A659G:p.Y220C,TP53:NM_001276695:exon6:c.A542G:p.Y181C,TP53:NM_001276696:exon6:c.A542G:p.Y181C,TP53:NM_001276760:exon6:c.A542G:p.Y181C,TP53:NM_001276761:exon6:c.A542G:p.Y181C
132		Wild type
7		TP53;U2AF1:NM_001025203:exon2:c.C101T:p.S34F U2AF1:NM_001025203:exon6:c.A470G:p.Q157R SETBP1:NM_015559:exon4:c.G2602A:p.D868N
139	exon12:c.C5885T:p.P1962L exon3:c.T3465C:p.N1155K	SETD2
65		ASXL1:NM_015338:exon12:c.C2077T:p.R693X U2AF1:NM_006758:exon2:c.C101T:p.S34F ETV6:NM_001987:exon7:c.T1193A:p.L398Q
162		Wild type
79		DNMT3A:NM_022552:exon23:c.G2645A:p.R882H
108		TET2:NM_001127208:exon11:c.A5284G:p.I1762V U2AF1:NM_006758:exon2:c.C101A:p.S34Y
187		DNMT3A:NM_022552:exon23:c.G2645A:p.R882H SRSF2:NM_003016:exon1:c.284_307del:p.95_103del

16		JAK2:NM_004972:exon14:c.G1849T:p.V617F<br>ASXL1:NM_015338:exon12:c.C1773A:p.Y591X
120		TET2:NM_001127208:exon11:c.A5284G:p.I1762V
141		Wild type
68		CEBPA:NM_004364:exon1:c.G175T:p.E59X<br>DNMT3A:NM_022552:exon23:c.G2645A:p.R882H<br>TET2:NM_001127208:exon3:c.C1207T:p.Q403X<br>U2AF1:NM_006758:exon2:c.C101T:p.S34F
22		DNMT3A:NM_022552:exon22:c.2479-1G>A
191		DNMT3A:NM_022552:exon23:c.G2645A:p.R882H<br>SRSF2:NM_003016:exon1:c.284_307del:p.95_103del
103		TET2:NM_001127208:exon11:c.A5284G:p.I1762V<br>ASXL1:NM_015338:exon12:c.2176_2177insAG:p.K726fs<br>U2AF1:NM_006758:exon2:c.C101T:p.S34F rs371769427
194	exon12:c.C5885T:p.P1962L<br>exon3:c.G3240A:p.M1080I	U2AF1:NM_006758:exon6:c.A470C:p.Q157P
14		Wild type
193		TP53:NM_000546:exon8:c.G818T:p.R273L<br>NRAS:NM_002524:exon2:c.G34A:p.G12S
26		ASXL1:NM_015338:exon12:c.1927dupG:p.G642fs<br>U2AF1:NM_006758:exon2:c.C101T:p.S34F<br>NRAS:NM_002524:exon2:c.G34T:p.G12C rs121913250
32		ASXL1:NM_015338:exon12:c.1927dupG:p.G642fs<br>U2AF1:NM_006758:exon2:c.C101T:p.S34F rs371769427
99		RUNX1:NM_001754:exon6:c.601delC:p.R201fs<br>TET2:NM_001127208:exon3:c.3340dupA:p.D1113fs
136	exon12:c.C5885T:p.P1962L	KMT2A:NM_001197104:exon1:c.C92A:p.P31Q
94		TET2:NM_001127208:exon11:c.A5284G:p.I1762V
28		ASXL1:NM_015338:exon12:c.1927delG:p.G643fs
77		NRAS:NM_002524:exon2:c.G37C:p.G13R<br>KRAS:NM_004985:exon2:c.G35C:p.G12A<br>U2AF1:NM_006758:exon2:c.C101T:p.S34F
143		SETD2

78		NPM1:NM_002520:exon11:c.859_860insTCTG:p.L287fs<br>FLT3-ITD:NM_004119:exon14:c.1800_1801insTACTTCTACGTTGATTTCAGAGAATATGAATATGAT:p.L601delinsYFYVDFREYEYDL
48		SF3B1:NM_012433:exon14:c.G1998T:p.K666N<br>JAK2:NM_004972:exon14:c.G1849T:p.V617F<br>KMT2A:NM_001197104:exon27:c.G10093A:p.V3365I
184		SETD2
145		JAK2:NM_004972:exon14:c.G1849T:p.V617F<br>SRSF2:NM_003016:exon1:c.C284A:p.P95H<br>TET2:NM_001127208:exon3:c.3340dupA:p.D1113fs
146		U2AF1:NM_006758:exon2:c.C101A:p.S34Y
56		ASXL1:NM_015338:exon12:c.G3671C:p.R1224T<br>ASXL1:NM_015338:exon12:c.C1815A:p.C605X<br>CBL:NM_005188:exon8:c.T1111C:p.Y371H<br>JAK2:NM_004972:exon14:c.G1849T:p.V617F
163		Wild type
45		TP53:NM_000546:exon7:c.G725A:p.C242Y
164		TP53:NM_000546:exon5:c.C476T:p.A159V<br>TET2:NM_001127208:exon11:c.A5284G:p.I1762V
128		Wild type
186		DNMT3A:NM_022552:exon23:c.G2645A:p.R882H
73		SF3B1:NM_012433:exon14:c.C1873T:p.R625C
137	exon12:c.C5885T:p.P1962L	SRSF2:NM_003016:exon1:c.C284A:p.P95H<br>SF3B1:NM_012433:exon14:c.G1998T:p.K666N
3-C		SRSF2:NM_003016:exon1:c.C284A:p.P95H<br>TP53:NM_000546:exon5:c.C476T:p.A159V<br>DNMT3A:NM_022552:exon23:c.G2645A:p.R882H
21		NPM1:NM_002520:exon11:c.859_860insTCTG:p.L287fs<br>DNMT3A:NM_022552:exon23:c.G2645A:p.R882H<br>ASXL1:NM_015338:exon12:c.A2648G:p.E883G
104		IDH2:NM_002168:exon4:c.G419A:p.R140Q<br>TET2:NM_001127208:exon11:c.5180_5184del:p.H1727fs<br>ASXL1:NM_015338:exon12:c.1927dupG:p.G642fs<br>SRSF2:NM_003016:exon1:c.C284A:p.P95H
33		ZRSR2:NM_005089:exon5:c.C328T:p.Q110X<br>ASXL1:NM_015338:exon12:c.3480_3481insATGGTTG:p.G1160fs
179		TET2:NM_001127208:exon11:c.5180_5184del:p.H1727fs

17		IDH2:NM_002168:exon4:c.G419A:p.R140Q<br>SF3B1:NM_012433:exon14:c.A1997G:p.K666R
172		Wild type
203		SRSF2:NM_003016:exon1:c.C284A:p.P95H<br>TET2:NM_001127208:exon3:c.3340dupA:p.D1113fs<br>DNMT3A:NM_022552:exon23:c.G2645A:p.R882H<br>ASXL1:NM_015338:exon12:c.A2648G:p.E883G
36		SRSF2:NM_003016:exon1:c.C284A:p.P95H<br>ASXL1:NM_015338:exon12:c.3036_3046del:p.D1012fs<br>TET2:NM_001127208:exon3:c.G3009A:p.W1003X
196		DNMT3A:NM_022552:exon23:c.G2645A:p.R882H
82		TET2:NM_001127208:exon11:c.A5284G:p.I1762V<br>KRAS:NM_004985:exon3:c.C173T:p.T58I<br>ASXL1:NM_015338:exon12:c.1927dupG:p.G642fs<br>U2AF1:NM_006758:exon6:c.A470C:p.Q157P<br>ETV6:NM_001987:exon3:c.C313G:p.R105G
160	exon3:c.C578T:p.P193L,exon3:c.G3240A:p.M1080I	SRSF2:NM_003016:exon1:c.C284A:p.P95H<br>TP53:NM_000546:exon5:c.C476T:p.A159V
46	exon12:c.C5885T:p.P1962L<br>exon3:c.T3465C:p.N1155K	CBL:NM_005188:exon8:c.T1186C:p.C396R<br>PHF6:NM_001015877:exon5:c.379_380del:p.Y127fs<br>TET2:NM_001127208:exon7:c.G3893A:p.C1298Y<br>TET2:NM_001127208:exon3:c.2916delT:p.S972fs<br>TET2:NM_001127208:exon3:c.C1648T:p.R550X
119		TET2：NM_001127208:exon11:c.A5284G:p.I1762V
192		DNMT3A:NM_022552:exon23:c.G2645A:p.R882H
147		Wild type
114		TET2：NM_001127208:exon11:c.A5284G:p.I1762V
72		IDH1:NM_005896:exon4:c.C394T:p.R132C<br>ASXL1:NM_015338:exon12:c.1927dupG:p.G642fs
92	exon3:c.G3240A:p.M1080I<br>exon12:c.C5885T:p.P1962L;SETD2:NM_014159:exon17c.7162del	TP53:NM_000546:exon5:c.C476T:p.A159V

	A;p.T2388fsX	
127		Wild type
11		U2AF1:NM_006758:exon2:c.C101T:p.S34F
97		DNMT3A:NM_022552:exon23:c.G2645A:p.R882H<br>TET2:NM_001127208:exon11:c.A5284G:p.I1762V<br>TET2:NM_001127208:exon3:c.G652A:p.V218M<br>BCOR:NM_001123384:exon12:c.4659_4662del:p.V1553fs<br>SF3B1:NM_012433:exon14:c.G1998C:p.K666N
30		Wild type
113		TP53:NM_000546:exon7:c.C722T:p.S241F;TET2:NM_001127208:exon11:c.A5284G:p.I1762V<br>ASXL1:NM_015338:exon12:c.2323delT:p.L775X<br>SRSF2:NM_003016:exon1:c.C283G:p.P95A<br>CBL:NM_005188:exon9:c.G1259A:p.R420Q
12		MPL:NM_005373:exon10:c.1543_1544TG>AA:p.W515K<br>ASXL1:NM_015338:exon12:c.T2761C:p.S921P
169		Wild type
9		NRAS:NM_002524:exon3:c.C181A:p.Q61K<br>KRAS:NM_004985:exon2:c.G37T:p.G13C rs121913535
61		RUNX1:NM_001754:exon6:c.601delC:p.R201fs
10	exon3:c.G3240A:p.M1080I<br>exon3:c.T3465C:p.N1155K	ASXL1:NM_015338:exon12:c.1927dupG:p.G642fs<br>SRSF2:NM_003016:exon1:c.C284A:p.P95H
95	exon12:c.C5885T:p.P1962L	DNMT3A:NM_022552:exon19:c.G2207A:p.R736H<br>TET2:NM_001127208:exon11:c.A5284G:p.I1762V<br>TP53:NM_000546:exon7:c.C722T:p.S241F rs28934573
3		NRAS:NM_002524:exon3:c.C181A:p.Q61K rs121913254<br>FLT3-ITDins≈65bp
161		Wild type
111		TET2:NM_001127208:exon11:c.A5284G:p.I1762V
102		U2AF1:NM_006758:exon2:c.C101T:p.S34F rs371769427<br>ASXL1:NM_015338:exon12:c.G1748A:p.W583X
2-C	exon12:c.C5885T:p.P1962L<br>exon3:c.T3465C:p.N1155K	

81		TET2:NM_001127208:exon3:c.G652A:p.V218M<br>TET2:NM_001127208:exon11:c.A5284G:p.I1762V<br>ASXL1:NM_015338:exon12:c.2216delT:p.L739fs<br>U2AF1:NM_006758:exon2:c.C101T:p.S34F<br>U2AF1:NM_006758:exon6:c.A470G:p.Q157R<br>JAK2:NM_004972:exon14:c.G1849T:p.V617F
168		Wild type
197		DNMT3A:NM_022552:exon23:c.C2644T:p.R882C<br>ASXL1:NM_015338:exon12:c.2216delT:p.L739fs
51		NPM1:NM_002520:exon11:c.859_860insTCTG:p.L287fs<br>DNMT3A:NM_022552:exon23:c.G2645A:p.R882H<br>FLT3:NM_004119:intron14:chr13:28608214_28608215insATG GGAGTTTCCAAGAGAAAATTTAGAGTTTGGTAA
15		TET2:NM_001127208:exon11:c.5132delT:p.I1711fs<br>TET2:NM_001127208:exon6:c.3765delC:p.Y1255X<br>CBL:NM_005188:exon8:c.T1111G:p.Y371D<br>SETBP1:NM_015559:exon4:c.A2621T:p.D874V
38		TP53:NM_000546:exon7:c.T770C:p.L257P<br>ZRSR2:NM_005089:exon11:c.1338_1339insAGCCGC:p.R446 delinsRSR
40		DNMT3A:NM_022552:exon23:c.C2644T:p.R882C<br>IDH2:NM_002168:exon4:c.G419A:p.R140Q<br>NRAS:NM_002524:exon2:c.G35A:p.G12D
124		Wild type
13		Wild type
159		Wild type
116		TET2:NM_001127208:exon3:c.G652A:p.V218M rs6843141
6		SF3B1:NM_012433:exon14:c.G1998C:p.K666N<br>NRAS:NM_002524:exon2:c.G34A:p.G12S<br>IDH1:NM_005896:exon4:c.G395A:p.R132H<br>IDH2:NM_002168:exon4:c.G419A:p.R140Q<br>NPM1:NM_002520:exon11:c.860_861insCTGC:p.L287fs
64		SETBP1:NM_015559:exon4:c.T2612C:p.I871T
174	exon12:c.C5885 T:p.P1962L<br>exon3:c.T3465C: p.N1155K	SETD2
129		Wild type

5		U2AF1:NM_001025203:exon2:c.C101T:p.S34F<br>CEBPA:NM_004364:exon1:c.1067dupA:p.N356fs
155		U2AF1:NM_001025203:exon2:c.C101T:p.S34F<br>CEBPA:NM_004364:exon1:c.A1060C:p.M354L<br>CEBPA:NM_004364:exon1:c.1067dupA:p.N356fs
96	exon12:c.C5885T:p.P1962L<br>exon3:c.T3465C:p.N1155K	KRAS:NM_004985:exon2:c.G38A:p.G13D
165		Wild type
101		FLT3-ITD:NM_004119:exon14c.1798_1799<br>insCCGGCTCCTCAGATAATGAGTACTTCTACGTTGATTTCAGAGAATATGAATATG:<br>p.D600delinsAGSSDNEYFYVDFREYEYD<br>NPM1:NM_002520:exon11:c.859_860insTCTG:p.L287fs<br>DNMT3A:NM_022552:exon23:c.G2645A:p.R882H<br>TET2:NM_001127208:exon7:c.3812dupG:p.C1271fs
23		TP53:NM_000546:exon8:c.G818A:p.R273H
126		Wild type
25		ASXL1:NM_015338:exon12:c.C2074T:p.Q692X<br>EZH2:NM_004456:exon8:c.T898C:p.C300R.
167		Wild type
183		SRSF2:NM_003016:exon1:c.C284A:p.P95H<br>DNMT3A:NM_022552:exon23:c.G2645A:p.R882H
71		FLT3:NM_004119:exon20:c.A2516G:p.D839G<br>NPM1:NM_002520:exon11:c.859_860insTCTG:p.L287fs<br>NRAS:NM_002524:exon2:c.G35A:p.G12D<br>NRAS:NM_002524:exon2:c.G38A:p.G13D
2		IDH1:NM_005896:exon4:c.G395A:p.R132H<br>TET2:NM_001127208:exon3:c.C2791T:p.P931S
133		Wild type
83		TET2:NM_001127208:exon11:c.A5284G:p.I1762V<br>ASXL1:NM_015338:exon12:c.2583dupA:p.A861fs<br>U2AF1:NM_006758:exon2:c.C101A:p.S34Y
173		NRAS:NM_002524:exon2:c.G38A:p.G13D
201		TP53:NM_000546:exon8:c.G818A:p.R273H<br>SRSF2:NM_003016:exon1:c.C284A:p.P95H<br>TP53:NM_000546:exon5:c.C476T:p.A159V<br>DNMT3A:NM_022552:exon23:c.G2645A:p.R882H

69	exon12:c.C5885T:p.P1962L<br>exon3:c.T3465C:p.N1155K	SRSF2:NM_003016:exon1:c.C284A:p.P95H<br>CBL:NM_005188:exon8:c.A1193C:p.H398P<br>CBL:NM_005188:exon9:c.C1258T:p.R420X<br>TET2:NM_001127208:exon3:c.818delA:p.Q273fs<br>JAK2:NM_004972:exon14:c.G1849T:p.V617F<br>CBL:NM_005188:exon8:c.A1169T:p.D390V
50		NRAS:NM_002524:exon2:c.G35A:p.G12D<br>NPM1:NM_002520:exon11:c.861_862insTGCA:p.L287fs<br>FLT3-ITD:NM_004119:exon14:c.1778_1779insACAGGTGACCGGCTCCTCAGATAATGAGTACTTCTACGTTGA:p.D593delinsEQVTGSSDNEYFYVD<br>DNMT3A:NM_022552:exon8:c.C920T:p.P307L
54		CBL:NM_005188:exon8:c.T1150C:p.C384R<br>SRSF2:NM_003016:exon1:c.C284G:p.P95R
110		TET2:NM_001127208:exon11:c.A5284G:p.I1762V<br>RUNX1:NM_001754:exon4:c.119_173del:p.F40fs<br>RUNX1:NM_001754:exon7:c.676_683del:p.S226fs<br>U2AF1:NM_006758:exon2:c.C101T:p.S34F<br>ETV6:NM_001987:exon6:c.1100_1102del:p.367_368del
29		TET2:NM_001127208:exon11:c.A5650G:p.T1884A<br>SRSF2:NM_003016:exon1:c.C284A:p.P95H
90		TET2:NM_001127208:exon3:c.C2290T:p.Q764X<br>TET2:NM_001127208:exon11:c.4947delT:p.Y1649fs<br>BCOR:NM_001123384:exon7:c.C3493T:p.R1165X
156		DNMT3A:NM_022552:exon16:c.G1906A:p.V636M<br>IDH2:NM_002168:exon4:c.G515A:p.R172K<br>BCOR:NM_001123383:exon4:c.2811delC:p.P937fs<br>NRAS:NM_002524:exon2:c.G35A:p.G12D
31	exon12:c.C5885T:p.P1962L<br>exon3:c.T3465C:p.N1155K<br>exon3:c.C578T:p.P193L	TP53:NM_000546:exon5:c.C406T:p.Q136X
18		SF3B1:NM_012433:exon15:c.A2098G:p.K700E
67		SF3B1:NM_012433:exon15:c.A2098G:p.K700E
153		Wild type
39		RUNX1:NM_001754:exon6:c.C610T:p.R204X<br>U2AF1:NM_006758:exon6:c.A470C:p.Q157P<br>TET2:NM_001127208:exon5:c.G3578A:p.C1193Y

57	exon12:c.C5885T:p.P1962L	TP53:NM_000546:exon5:c.T526C:p.C176R<br>JAK2:NM_004972:exon14:c.G1849T:p.V617F<br>DNMT3A:NM_022552:exon20:c.C2391G:p.N797K
100	exon3:c.G2283A:p.M761I	SETD2
199		TP53:NM_000546:exon7:c.C722T:p.S241F;DNMT3A:NM_022552:exon16:c.G1906A:p.V636M;TET2:NM_001127208:exon11:c.A5284G:p.I1762V rs2454206<br>SRSF2:NM_003016:exon1:c.C283G:p.P95A<br>SF3B1:NM_012433:exon15:c.A2098G:p.K700E
8		ASXL1:NM_015338:exon12:c.C4243T:p.R1415X<br>SF3B1:NM_012433:exon14:c.G1866T:p.E622D<br>TET2:NM_001127208:exon3:c.C1975T:p.Q659X
122		FLT3-ITD：NM_004119:exon14:c.1804_1805insGTGATTTCAGAGAATATGAATATGATCTCA:p.K602delinsSDFREYEYDLK<br>NPM1：NM_002520:exon11:c.859_860insTCTG:p.L287fs<br>DNMT3A：NM_022552:exon23:c.G2645A:p.R882H<br>TET2：NM_001127208:exon11:c.A5284G:p.I1762V
1		ASXL1:NM_015338:exon12:c.C1774T:p.Q592X
149		ASXL1:NM_015338:exon12:c.C1774T:p.Q592X
144		Wild type
121		TET2：NM_001127208:exon11:c.A5284G:p.I1762V<br>U2AF1：NM_006758:exon2:c.C101T:p.S34F
76		U2AF1:NM_006758:exon2:c.C101T:p.S34F<br>SETBP1:NM_015559:exon4:c.G2608A:p.G870S
49		TET2:NM_001127208:exon3:c.C1642T:p.Q548X<br>SF3B1:NM_012433:exon15:c.A2098G:p.K700E
60	exon12:c.C5885T:p.P1962L	TP53:NM_000546:exon6:c.C569T:p.P190L
198		RUNX1:NM_001754:exon6:c.548_549insCC:p.P183fs
35		TP53:NM_000546:exon7:c.T761C:p.I254T
118		TET2:NM_001127208:exon11:c.4706delA:p.Y1569fs<br>ASXL1:NM_015338:exon12:c.2323delT:p.L775X

		IDH2:NM_002168:exon4:c.G419A:p.R140Q<br>NRAS:NM_002524:exon2:c.G38A:p.G13D
87		U2AF1:NM_006758:exon2:c.C101T:p.S34F
142		Wild type
148		TET2:NM_001127208:exon3:c.219dupT:p.R73fs
190		DNMT3A:NM_022552:exon20:c.C2391G:p.N797K;SRSF2:NM_003016:exon1:c.C284A:p.P95H ;SF3B1:NM_012433:exon15:c.A2098G:p.K700E;U2AF1:NM_006758:exon2:c.C101T:p.S34F
112		ASXL1:NM_015338:exon12:c.2323delT:p.L775X
88		MPL:NM_005373:exon6:c.G962A:p.R321Q
109		CEBPA:NM_004364:exon1:c.A1060C:p.M354L<br>TET2:NM_001127208:exon11:c.A5284G:p.I1762V<br>TET2:NM_001127208:exon3:c.C3058T:p.Q1020X<br>TET2:NM_001127208:exon3:c.1726_1727insAATCCCA:p.E576fs<br>U2AF1:NM_006758:exon2:c.C101T:p.S34F
200		ASXL1:NM_015338:exon12:c.C4243T:p.R1415X<br>ADNMT3A:NM_022552:exon20:c.C2391G:p.N797K
62	exon12:c.C5885T:p.P1962L	TP53:NM_000546:exon7:c.C722G:p.S241C
91		TET2:NM_001127208:exon11:c.A5284G:p.I1762V
202		DNMT3A:NM_022552:exon20:c.C2391G:p.N797K/SRSF2:NM_003016:exon1:c.C284A:p.P95H<br>SF3B1:NM_012433:exon15:c.A2098G:p.K700E
117		NPM1:NM_002520:exon11:c.859_860insTCTG:p.L287fs<br>IDH2:NM_002168:exon4:c.G419A:p.R140Q<br>PHF6:NM_001015877:exon10:c.C1024T:p.R342X<br>NRAS:NM_002524:exon2:c.G34A:p.G12S
185		DNMT3A:NM_022552:exon16:c.G1906A:p.V636M
152		Wild type
43		SF3B1:NM_012433:exon15:c.A2098G:p.K700E<br>TP53:NM_000546:exon7:c.G775T:p.D259Y
106		TET2:NM_001127208:exon11:c.A5284G:p.I1762V
105	exon12:c.C5885T:p.P1962L	TET2:NM_001127208:exon11:c.A5284G:p.I1762V<br>TP53:NM_000546:exon8:c.C916T:p.R306X

75		U2AF1:NM_006758:exon2:c.C101T:p.S34F
4	exon12:c.C5885T:p.P1962L	DNMT3A:NM_022552:exon23:c.G2645A:p.R882H<br>NRAS:NM_002524:exon2:c.G38A:p.G13D<br>KRAS:NM_004985:exon2:c.G35A:p.G12D
189		DNMT3A:NM_022552:exon20:c.C2391G:p.N797K/SRSF2:NM_003016:exon1:c.C283G:p.P95A<br>SF3B1:NM_012433:exon15:c.A2098G:p.K700E<br>U2AF1:NM_006758:exon2:c.C101A:p.S34Y
47		SF3B1:NM_012433:exon15:c.A2098G:p.K700E<br>NRAS:NM_002524:exon2:c.G35A:p.G12D<br>ETV6:NM_001987:exon3:c.326dupC:p.S109fs
20		ASXL1:NM_015338:exon12:c.2295delG:p.L765fs<br>U2AF1:NM_006758:exon6:c.A470G:p.Q157R<br>U2AF1:NM_006758:exon2:c.C101T:p.S34F<br>NRAS:NM_002524:exon2:c.G34A:p.G12S<br>SETBP1:NM_015559:exon4:c.T2612C:p.I871T
24		CEBPA:NM_004364:exon1:c.954_955insGAGCTGACC:p.S319delinsELTS<br>TET2:NM_001127208:exon11:c.4706delA:p.Y1569fs
140		Wild type
34		TET2:NM_001127208:exon3:c.C86G:p.P29R rs12498609
66		CSF3R:NM_156039:exon17:c.C2213T:p.S738F
70		DNMT3A:NM_022552:exon8:c.C976A:p.R326S
182		DNMT3A:NM_022552:exon23:c.G2645A:p.R882H<br>SRSF2:NM_003016:exon1:c.C283G:p.P95A/<br>SF3B1:NM_012433:exon15:c.A2098G:p.K700E
19		U2AF1:NM_006758:exon2:c.C101A:p.S34Y
52	exon17:c.T7457G:p.L2486R	ASXL1:NM_015338:exon12:c.1888_1910del:p.H630fs<br>DNMT3A:NM_022552:exon23:c.G2645A:p.R882H<br>IDH2:NM_002168:exon4:c.G419A:p.R140Q<br>KRAS:NM_004985:exon2:c.G35C:p.G12A
80		TET2:NM_001127208:exon3:c.C86G:p.P29R<br>GATA2:NM_001145662:exon3:c.G490A:p.A164T
151		Wild type
89	exon3:c.G3240A:p.M1080I	SETD2
41		NPM1:NM_002520:exon11:c.859_860insTCTG:p.L287fs

130		Wild type

MATERIALS 2 Gene mutations-variants in MDS cases (somatic:negative or germline: positive)

SAMPLE 58 positive

RUNX1:NM_001754:exon6:c.G611A:p.R204Q (DNA of oral mucosal cells)

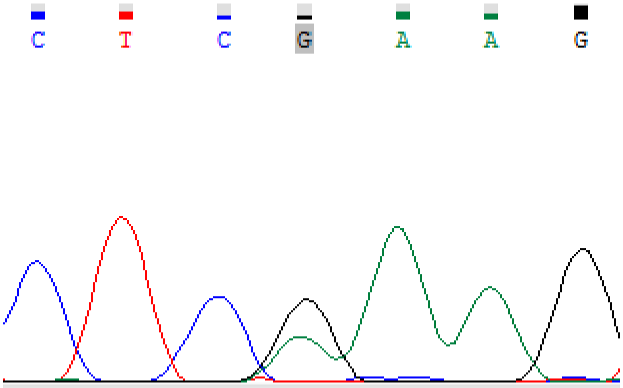

RUNX1:NM_001754:exon4:c.G281T:p.S94I

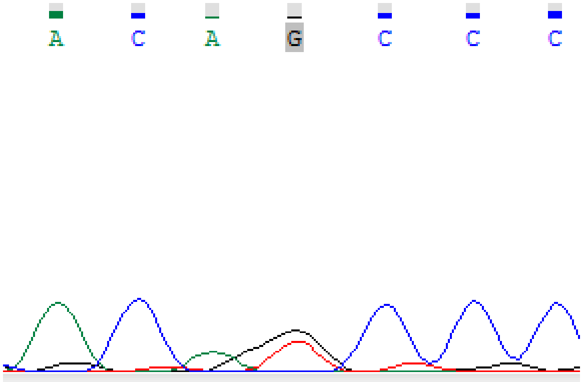

SAMPLE	c.G3240A M1080I	DNA of oral mucosal cells
55	GG	negative
37	GG	negative
194	GG	negative
160	GG	negative
92	GG	negative

10	GG	negative
89	GG	Negative

55

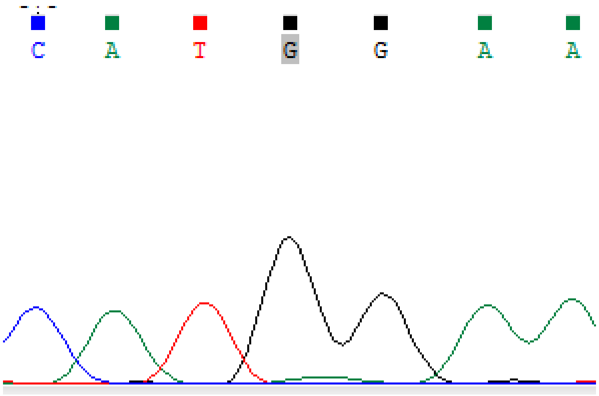

37

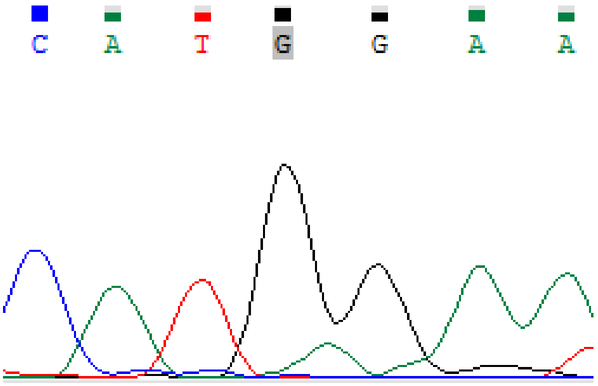

194

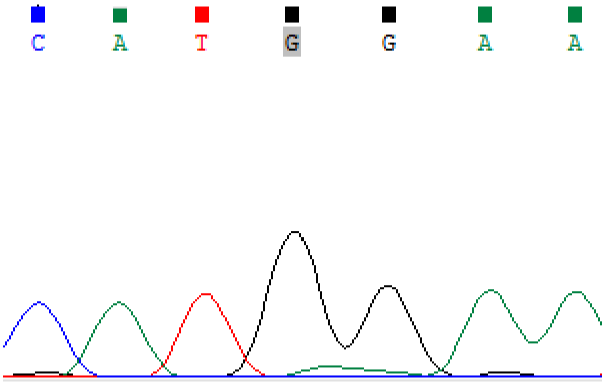

160

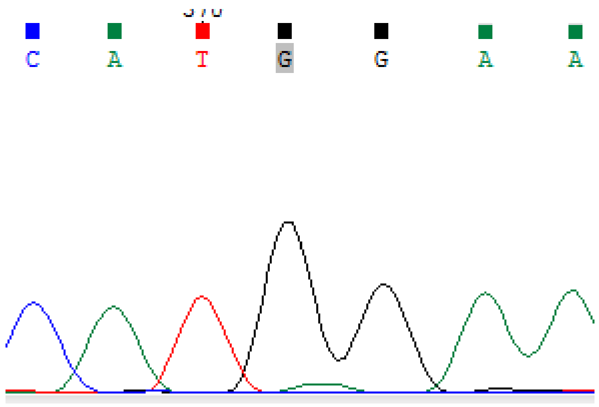

92

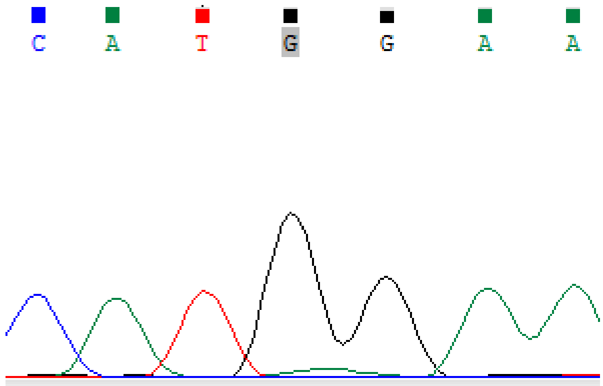

10

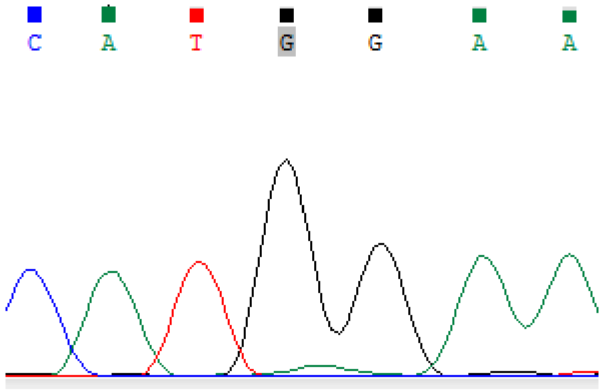

89

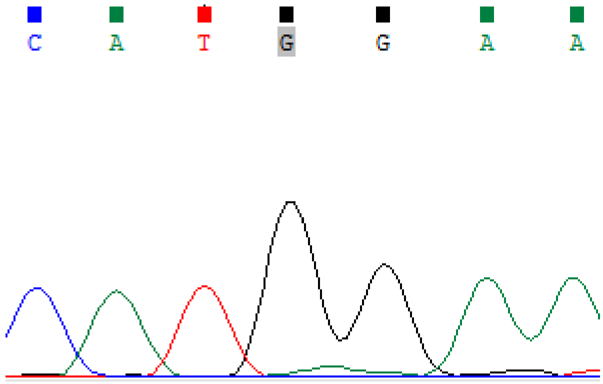

SAMPLE	c.G2283A M761I	DNA of oral mucosal cells
100	GG	negative

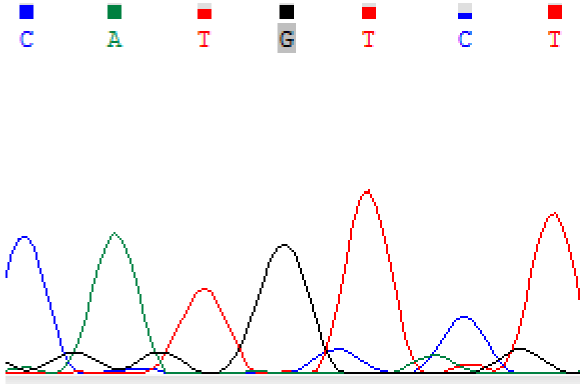

SAMPLE	c. G1915A E639K	DNA of oral mucosal cells
175	GG	negative
176	GG	negative

175

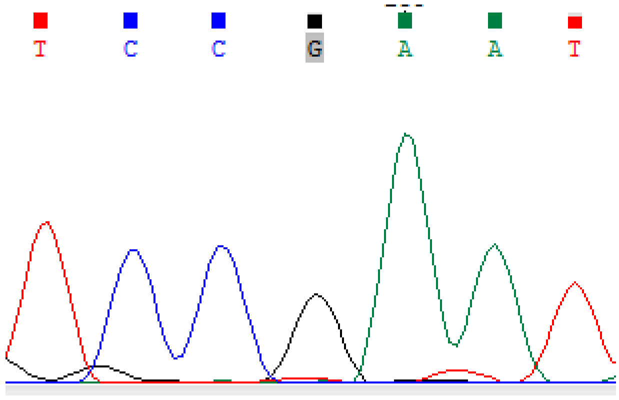

176

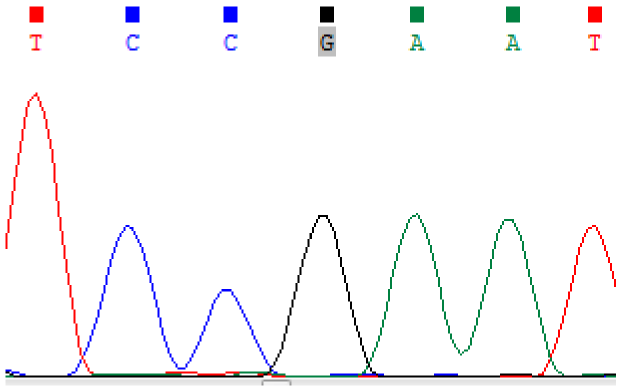

SAMPLE	E1142G	DNA of oral mucosal cells
178	GG	negative

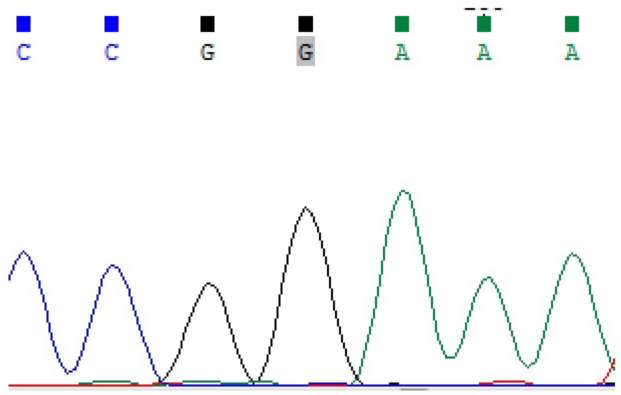

SAMPLE	c.C578T	DNA of oral mucosal cells
	P193L	
160	CC	negative
31	CC	negative

160

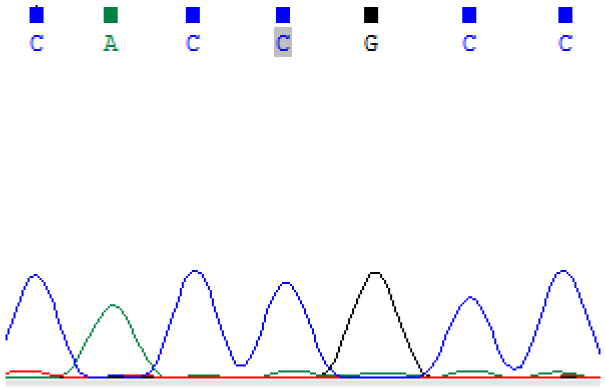

31

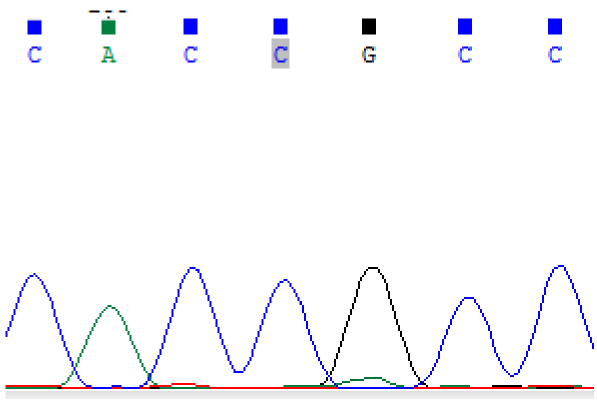

SAMPLE L2486R DNA of oral mucosal cells

L2486R

52 CTG negative

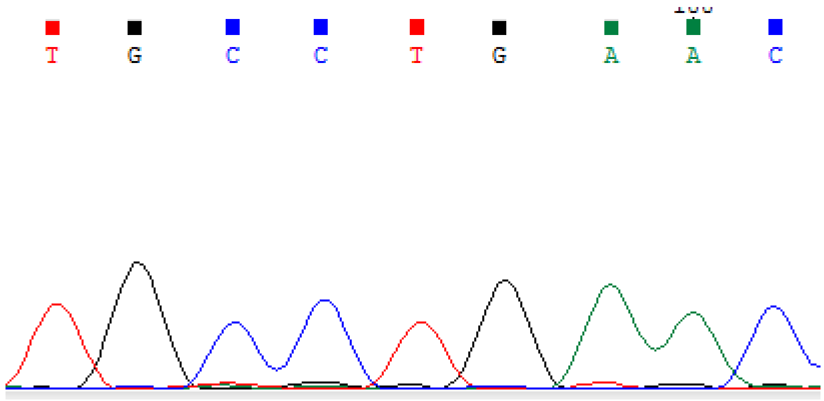

SAMPLE c.3350-3351insT DNA of oral mucosal cells

F1116fs

177 negative

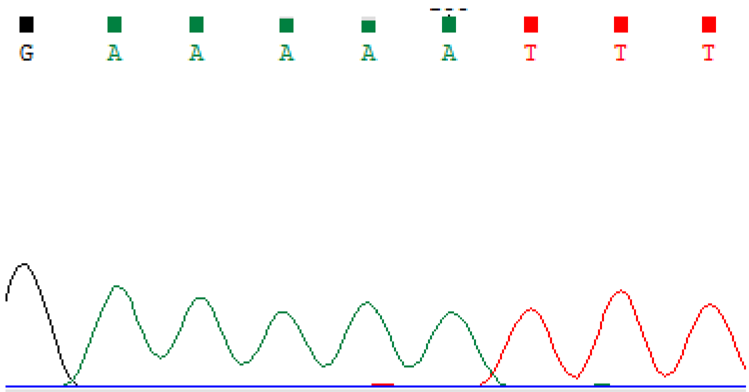

SAMPLE c.7162delA DNA of oral mucosal cells

T2388fs

92 negative

92-daughter negative

92

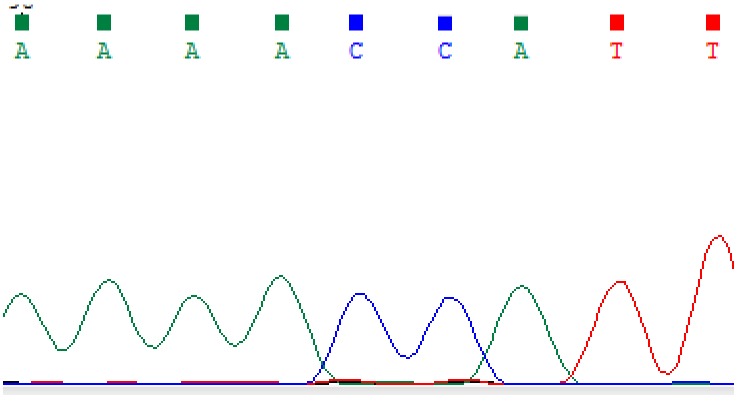

92-daughter

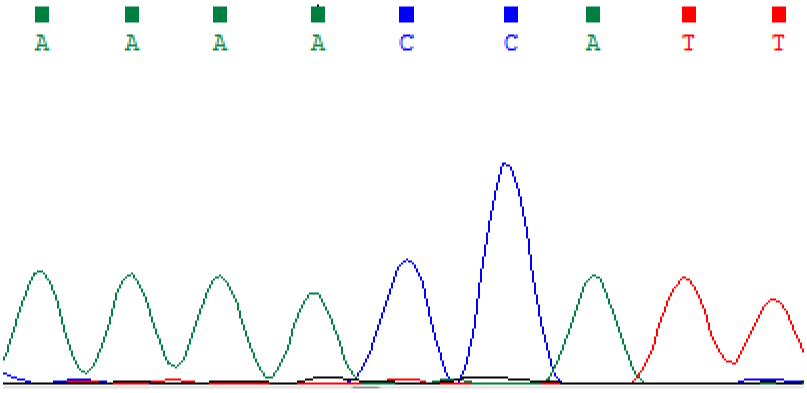

SAMPLE	c.C5885T p.(P1962L)	DNA of oral mucosal cells
69	CT	
37	CT	
59	TT	
58	TT	
44	CT	
4-C	CT	
84	TT	
55	CT	
92	CT	
31	TT	
46	TT	
123	CC	
42	CC	

69

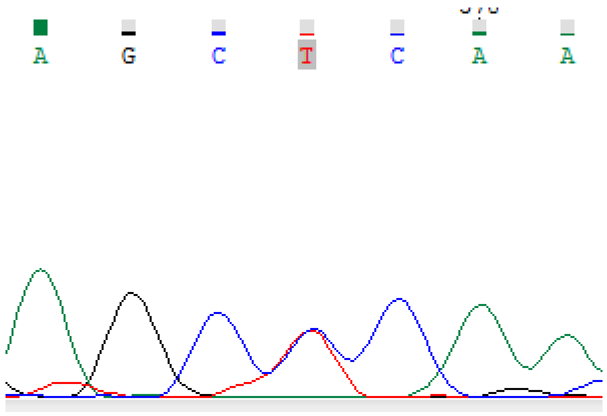

37

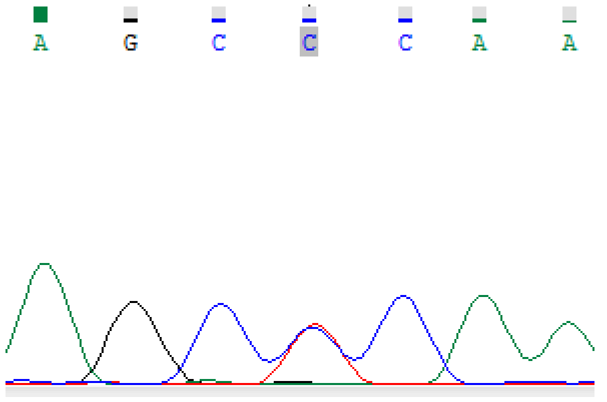

59

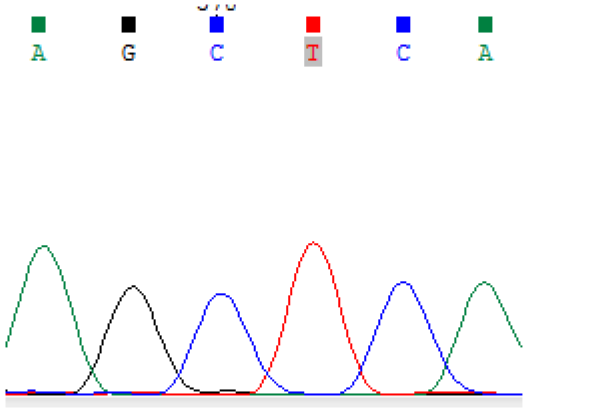

58

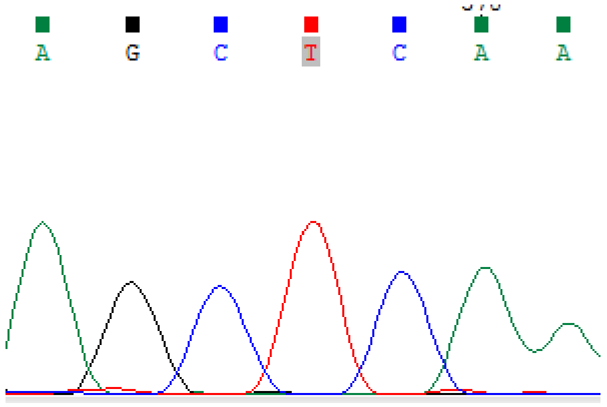

44

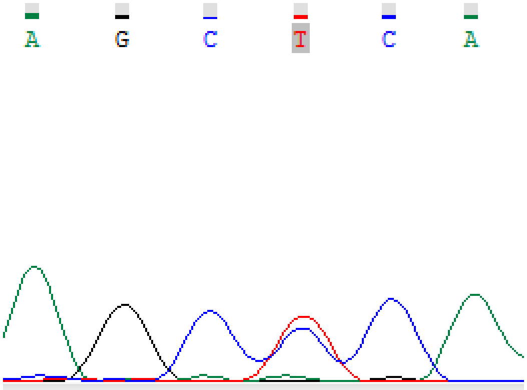

4-c

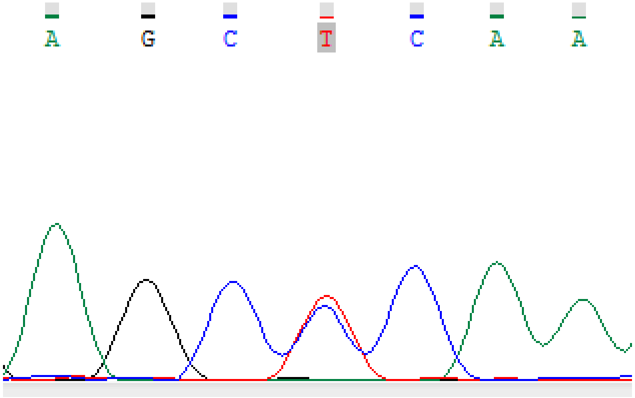

84

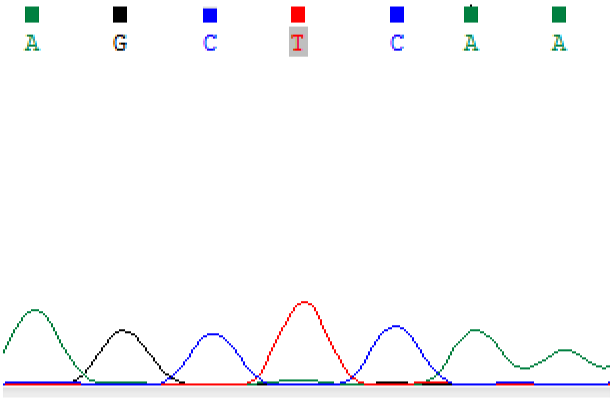

55

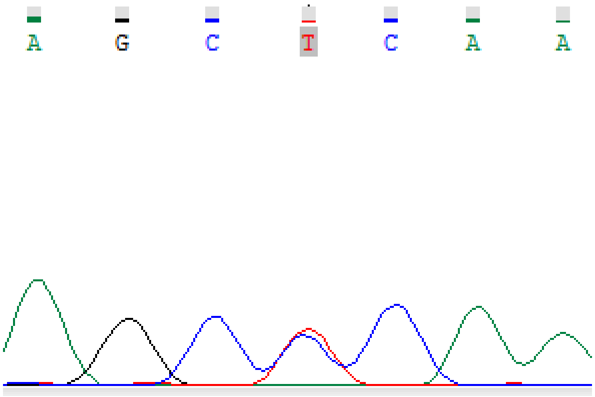

92

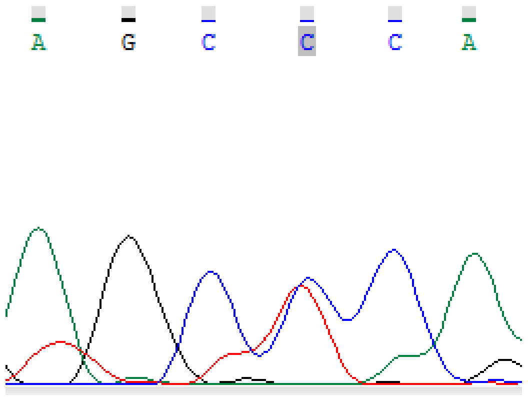

31

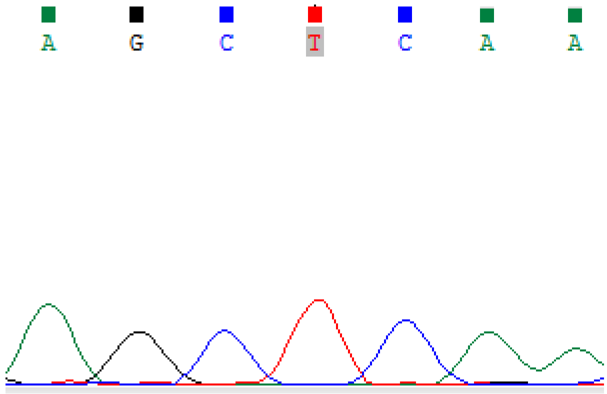

46

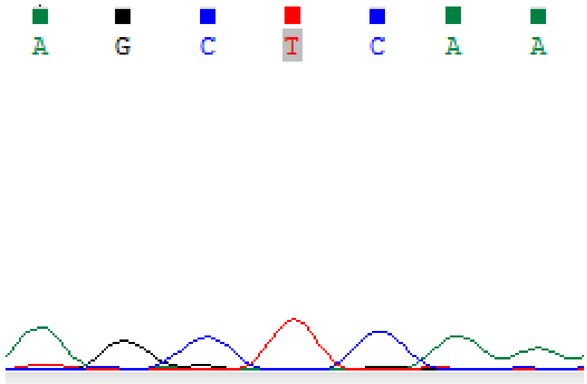

123

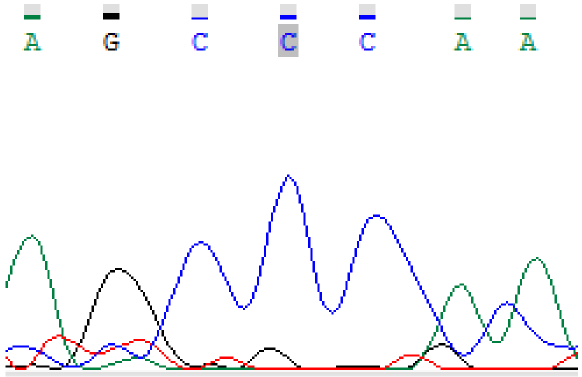

42

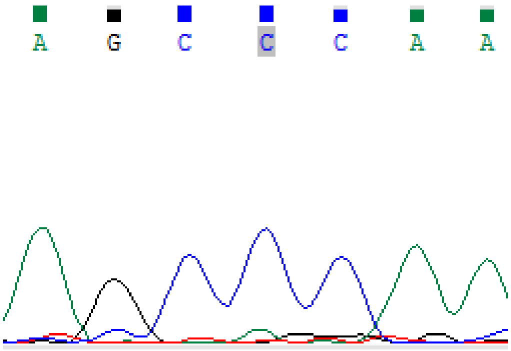

SAMPLE	c.T3465C N1155K	DNA of oral mucosal cells
42	TC	
58	CC	
44	CC	
84	TC	
4-C	CC	
46	CC	
55	TT	
138	TT	

42

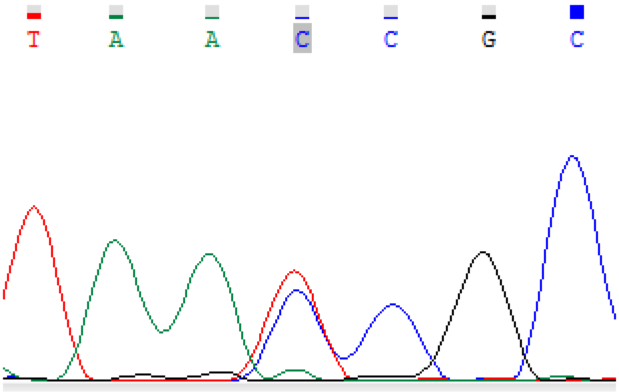

58

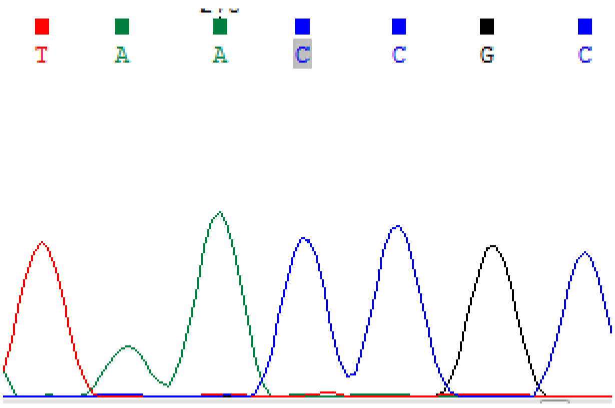

46

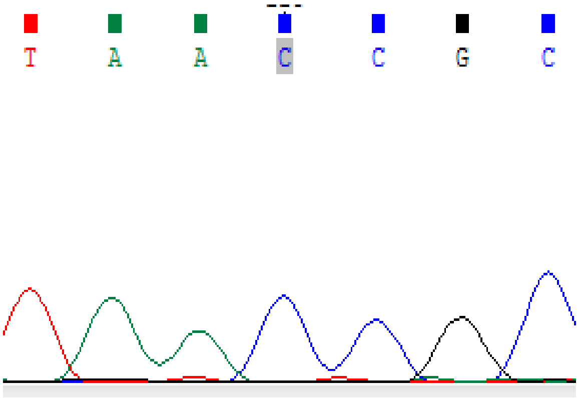

44

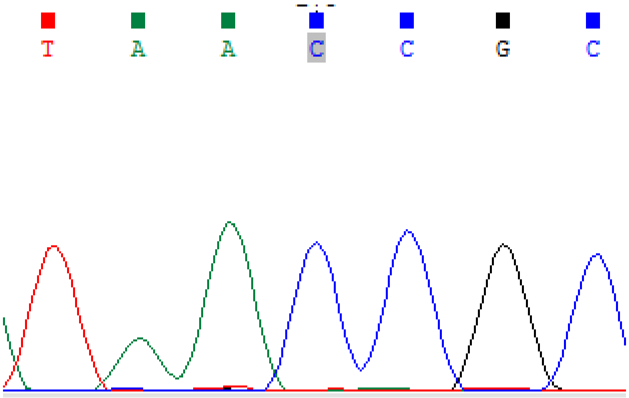

84

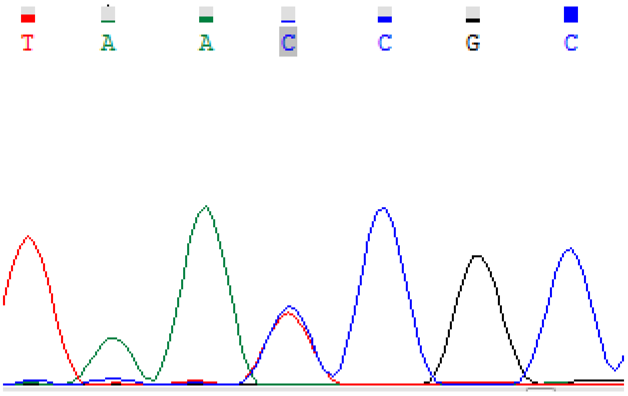

55

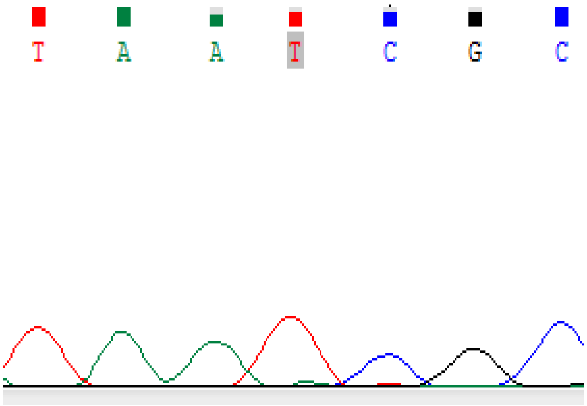

4-C

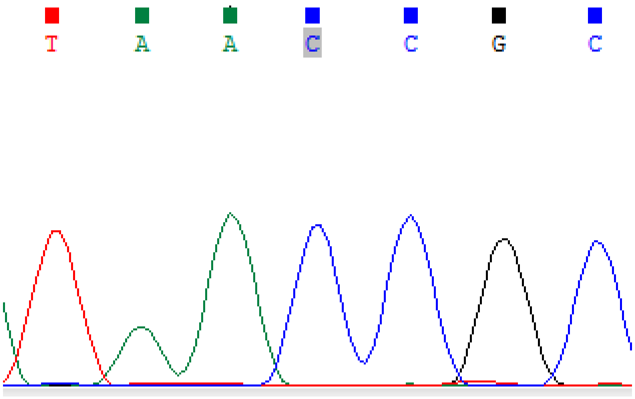

138

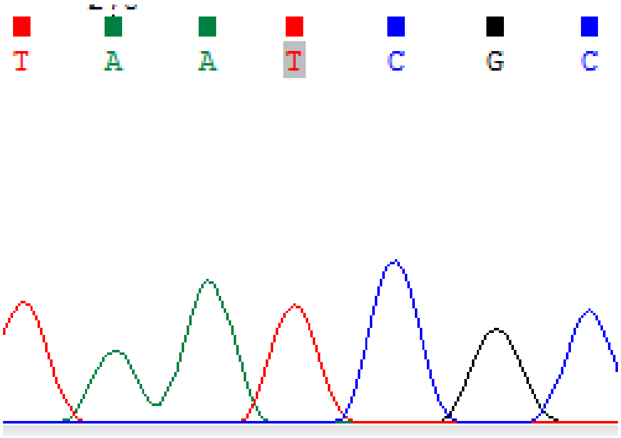

Supplement: Supplementary file 1 [file Presentation_1.pdf]
